# Delineating the Rules for Structural Adaptation of Membrane-Associated Proteins to Evolutionary Changes in Membrane Lipidome

**DOI:** 10.1016/j.cub.2019.11.043

**Published:** 2020-02-03

**Authors:** Maria Makarova, Maria Peter, Gabor Balogh, Attila Glatz, James I. MacRae, Nestor Lopez Mora, Paula Booth, Eugene Makeyev, Laszlo Vigh, Snezhana Oliferenko

**Affiliations:** 1 The Francis Crick Institute, 1 Midland Road, London NW1 1AT, UK; 2 Randall Centre for Cell and Molecular Biophysics, School of Basic and Medical Biosciences, King’s College London, Guy’s Campus, London SE1 1UL, UK; 3 Institute of Biochemistry, Biological Research Centre, Hungarian Academy of Sciences, Temesvári krt. 62, Szeged 6726, Hungary; 4 Department of Chemistry, King’s College London, Britannia House, London SE1 1DB, UK; 5 MRC Centre for Developmental Neurobiology, King’s College London, Guy’s Campus, London SE1 1UL, UK

**Keywords:** membranes, lipids, lipid metabolism, evolution, fission yeasts, adaptation, transmembrane proteins, fatty acid synthase, unfolded protein response

## Abstract

Membrane function is fundamental to life. Each species explores membrane lipid diversity within a genetically predefined range of possibilities. How membrane lipid composition in turn defines the functional space available for evolution of membrane-centered processes remains largely unknown. We address this fundamental question using related fission yeasts *Schizosaccharomyces pombe* and *Schizo*s*accharomyces japonicus*. We show that, unlike *S. pombe* that generates membranes where both glycerophospholipid acyl tails are predominantly 16–18 carbons long, *S. japonicus* synthesizes unusual “asymmetrical” glycerophospholipids where the tails differ in length by 6–8 carbons. This results in stiffer bilayers with distinct lipid packing properties. Retroengineered *S. pombe* synthesizing the *S.*-*japonicus*-type phospholipids exhibits unfolded protein response and downregulates secretion. Importantly, our protein sequence comparisons and domain swap experiments support the hypothesis that transmembrane helices co-evolve with membranes, suggesting that, on the evolutionary scale, changes in membrane lipid composition may necessitate extensive adaptation of the membrane-associated proteome.

## Highlights

• The two acyl tails in *S. japonicus* phospholipids tend to differ by 6–8 carbons
• *S. japonicus* but not *S. pombe* FAS makes both medium and long-chain fatty acids
• *S. japonicus* membranes are more ordered than membranes of its relative *S. pombe*
• Changes in membrane lipids may drive co-evolution of transmembrane helices

## Teaser

Makarova et al. show that membranes of related fission yeasts *S. pombe* and *S. japonicus* are made of structurally distinct phospholipids because of the difference in fatty acid synthase activities. Bioinformatics and retro-engineering experiments reveal that evolutionary changes in lipid metabolism require adaptation of the membrane-associated proteome.

## Introduction

Biological membranes are semi-permeable lipid barriers delimiting cells and subcellular compartments. By recruiting and scaffolding specific proteins and protein complexes, membranes serve as platforms for cellular communication, signaling, and metabolism. Structural and functional properties of membranes depend on their lipid composition [1, 2]. The main lipid classes found in membranes are the glycerophospholipids (GPLs), sphingolipids, and sterols. GPLs are further classified based on their variable polar head groups and hydrophobic fatty acyl (FA) tails, which can vary in length and saturation [3]. Membranes differ in lipid composition on multiple scales, suggesting that lipid diversity may be functionally consequential. Specific lipids may form nanoscale membrane domains, distribute asymmetrically between leaflets and show preferential enrichment in cellular organelles [4, 5]. Lipid diversity may contribute to differences in the physical properties of the lipid bilayer, such as hydrophobic thickness, lipid packing, and bending rigidity [5]. These in turn can affect the distribution and function of membrane-associated proteins [6]. Importantly, the lipid composition varies between species. Thus, understanding the physiological origins and role of membrane lipid diversity in cell biological processes is a fascinating problem that may also have important biomedical implications.

The fission yeasts *Schizosaccharomyces pombe* (*S. pombe*) and *Schizo*s*accharomyces japonicus* (*S. japonicus*) show remarkable differences in fundamental membrane-centered processes such as establishment of polarity and mitotic nuclear envelope (NE) remodeling [7, 8]. The different strategies of managing the NE have been linked to the distinct regulation of lipid synthesis during the cell cycle in the two species [9, 10].

## Results

### *S. pombe* and *S. japonicus* Exhibit Profound Differences in the Structure of Membrane Glycerophospholipids

**Figure 1:**
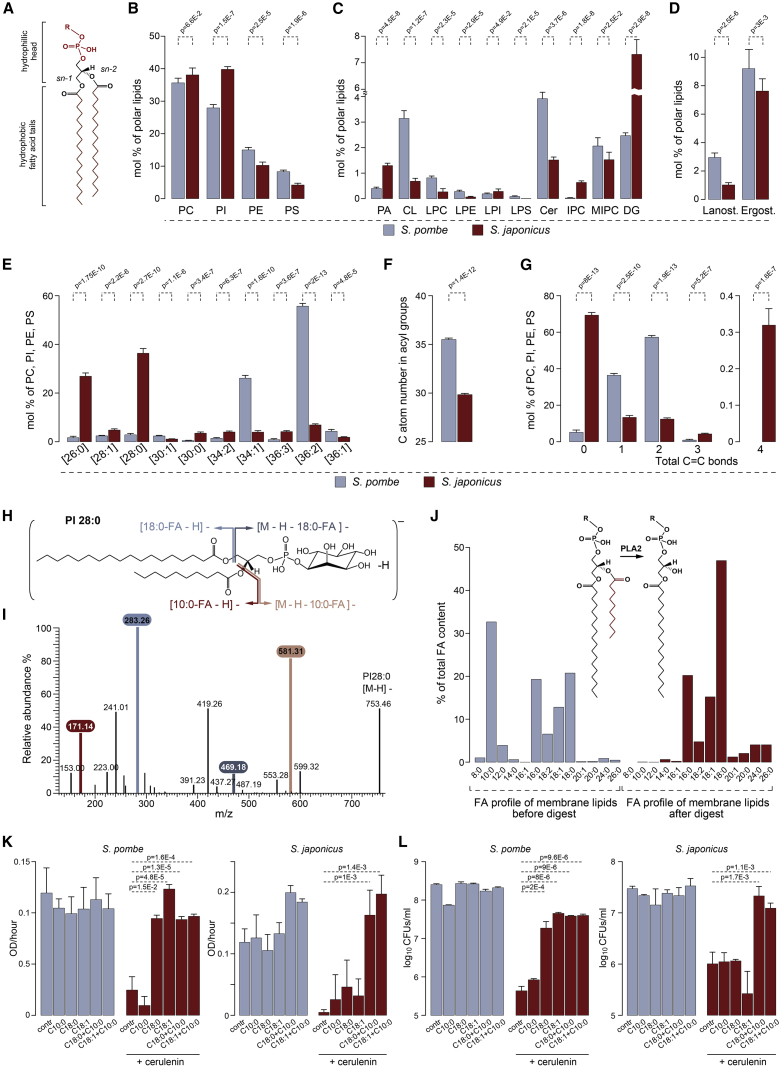
*S. japonicus* Relies on Production of Membrane Glycerophospholipids Exhibiting Pronounced Acyl Chain Asymmetry (A) Generic structure of a GPL molecule. (B) Relative abundance of the four main GPL classes (phosphatidylcholine (PC), phosphatidylinositol (PI), phosphatidylethanolamine (PE) and phosphatidylserine (PS)) in *S. pombe* and *S. japonicus*. (C) Relative abundance of the indicated lipid classes (phosphatidic acid (PA), cardiolipin (CL), lysophosphatidylcholine (LPC), lysophosphatidylethanolamine (LPE), lysophosphatidylinositol (LPI), lysophosphatidylserine (LPS), ceramide (Cer), inositol phosphoceramide (IPC), mannosyl-inositolphosphoceramide (MIPC), diacylglycerol (DG)) in *S. pombe* and *S. japonicus*. (D) Relative abundance of lanosterol and ergosterol in *S. pombe* and *S. japonicus*. (E) Molecular species composition calculated for the sum of PC, PI, PE, and PS in the two species. The categories shown are defined based on the total number of carbon atoms: total number of double bonds in acyls. (F) Average combined FA length in PC, PI, PE, and PS in the two species. (G) Comparison of FA saturation in PC, PI, PE, and PS in the two species. (H) A diagram of the 28:0 PI species abundant in *S. japonicus*. Colors and arrows indicate ions formed upon fragmentation. (I) A typical mass spectrum of the C28:0 PI species indicating ions generated upon fragmentation. Colors match the indicated fragments in (H). (J) Mass spectrometry analysis of FA content in GPLs before and after PLA2 treatment, which removes FAs specifically from the *sn*-2 position. Note that C10:0 is not recovered after digestion indicating that it is located at the *sn*-2 position. (K) Comparison of growth rates of *S. pombe* and *S. japonicus* cultures under indicated conditions. Growth rates were calculated for an exponential region of the growth curve as a change in OD_595_ per hour. (L) Survival of *S. pombe* and *S. japonicus* cells grown in Edinburgh minimal media (EMM) supplemented with indicated FAs loaded on BSA. Colony forming units (CFUs) per mL were counted after 48 h of growth. (B–G) Shown are the mean values ± SD (n = 5). p values are derived from the unpaired parametric t test. (K–L) Shown are the mean values ± SD (n = 3). p values are derived from the unpaired parametric t test. See also Figure S1 and Table S1.

To analyze membrane lipid compositions of the two sister species, we performed shotgun electrospray ionization tandem mass spectrometry (ESI-MS/MS) analysis of total cellular lipid extracts (Table 1 in Data S1). The most abundant membrane lipids were GPLs, defined by the polar head groups and FA chains at the *sn-1* and *sn-2* positions of the glycerol backbone (Figure 1A). We observed subtle differences in the abundance of four major GPL classes, including phosphatidylcholine (PC), phosphatidylethanolamine (PE), phosphatidylinositol (PI), and phosphatidylserine (PS) (Figure 1B). We also detected some variation in the abundance of the minor GPL classes, sphingolipids, sterols, and storage lipids, with *S. japonicus* containing less sterols and sphingolipids (Figures 1C, 1D, and S1A; Table 1 in Data S1).

Remarkably, we observed differences in the FA chain composition between *S. pombe* and *S. japonicus*. In line with previous reports, the most abundant molecular species in four major GPL classes in *S. pombe* were 36:2 and 34:1, where the first number indicates the combined length of FA tails and the second, total number of double bonds in FA tails (Figure 1E; Tables ii and iii in Data S1; [11]). The size profile of *S. pombe* GPLs was thus similar to the lipidome of the budding yeast *Saccharomyces cerevisiae* [12, 13]. However, *S. japonicus* GPLs exhibited a markedly distinct chemical composition, with abundant 26:0 and 28:0 molecular species (Figures 1E and 1F; Tables ii and iii in Data S1). Such a trend toward lower molecular weight species was present in every analyzed lipid class (Table 2 in Data S1).

In addition to differences in total length, phospholipid acyl chains were more saturated in *S. japonicus* compared to *S. pombe* (Figure 1G; Table 3 in Data S1). Despite this, we identified some polyunsaturated GPLs in *S. japonicus*, which were not detected in *S. pombe*, suggesting differences in desaturase activity (Figure 1G). This is consistent with the *S. japonicus* genome encoding a delta-12 desaturase, in addition to the delta-9 desaturase Ole1 [14], common to both fission yeasts and other eukaryotic groups [15].

In order to elucidate the structure of the lower molecular weight GPL species, we performed fragmentation analysis of the major GPL classes in *S. japonicus* (Figure 1H; Table S1). The resulting mass spectra indicated that C16 and C18 long-chain FAs were frequently found in combination with the medium-chain FA C10:0, forming asymmetrical structures, with C10:0 located in the *sn-2* position of the glycerol backbone (see data for an abundant PI species C28:0 in Figures 1H and 1I, with full interpretation of data in Table S1; data for the other major GPLs are presented in Table 4 in Data S1). We confirmed that C10:0 was found invariably in the *sn-2* position by analyzing the products of the *sn-2* specific phospholipase A2 (PLA2) digestion (Figure 1J). The proportion of such highly asymmetrical structures, where the two chains differed in length by 6–8 carbon atoms, varied between phospholipid classes, reaching ∼90% for PI (Figure S1B; Tables ii and iv in Data S1). We concluded that *S. japonicus* synthesizes a large proportion of highly asymmetrical GPLs.

To test whether C10:0 is essential for *S. japonicus* physiology, we inhibited endogenous FA synthesis with cerulenin [16] and supplemented both *S. pombe* and *S. japonicus* with exogenous FAs of different lengths and saturation. As expected, cell growth and survival of both species decreased drastically upon cerulenin treatment. The addition of exogenous long-chain fatty acids (LCFAs) such as oleic (C18:1) or stearic acid (C18:0) was sufficient to rescue both parameters in *S. pombe* (Figures 1K, 1L, and S1C). In the same experimental setup, LCFAs failed to rescue cerulenin-induced defects in growth and survival of *S. japonicus*. C10:0 alone was also insufficient, but, when it was added to cerulenin-treated *S. japonicus* cultures in combination with either C18:1 or C18:0, the growth and survival defects were largely rescued (Figures 1K, 1L, and S1D). When supplemented together with C18:1, C10:0 was 4.6 times more efficiently incorporated into GPLs of cerulenin-treated *S. japonicus* as compared with *S. pombe* (Figure S1E; Table 5 in Data S1). Together, these results demonstrate that *S. japonicus* physiology relies on the presence of both C10:0 and LCFAs.

### Lipid Packing and Membrane Rigidity Are Higher in Membranes Derived from *S. japonicus* Total Polar Lipids

**Figure 2:**
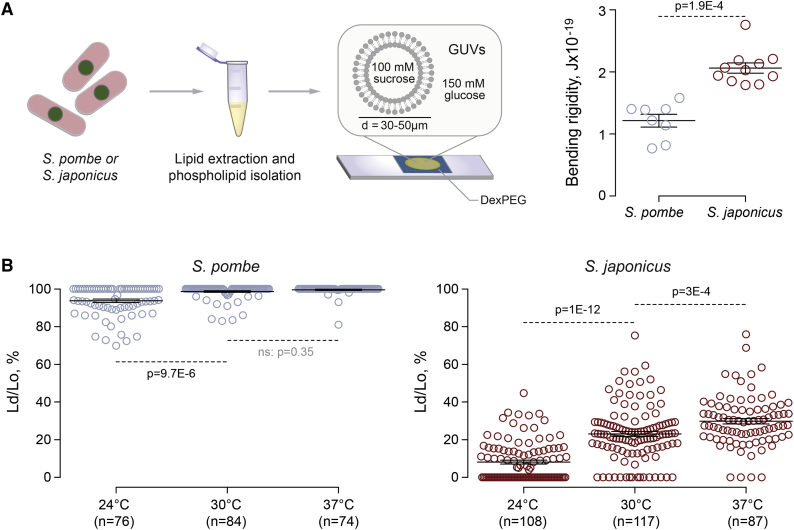
Membranes Assembled from *S. japonicus* Polar Lipids Exhibit Higher Membrane Stiffness and Lipid Packing (A) Shown on the left, in a typical experiment GUVs in a 30 to 50 μm range were obtained by hydrogel formation and thermal fluctuations were analyzed by light microscopy. Bending rigidities were calculated using fluctuation analysis. Shown on the right, one-dimensional scatterplot summarizing the results of these experiments. n = 8 and 11 for *S. pombe* and *S. japonicus*, respectively. (B) One-dimensional scatterplots summarizing the results of the temperature-dependent Lo and Ld phase separation in GUVs made of *S. pombe* (left) and *S. japonicus* (right) lipids. The Ld/Lo ratios were calculated from the measurements of the areas of distinct intensities at the middle plane of GUVs. Numbers of GUV measurements are indicated underneath each column. *In vitro* experiments were carried out with total polar lipid fractions. Means ± SD are indicated, and the p values are derived from the Kolmogorov-Smirnov test. See also Figure S2.

Differences in lipid structure may bear on biological functions by altering membrane’s properties. One of the fundamental biophysical parameters is the bending rigidity, which reflects how much energy is needed to deform the membrane from its intrinsic curvature. This energy reflects the bilayer stiffness [17]. We have generated giant unilamellar vesicles (GUVs), which are minimal systems to study the lipidic components of cellular membranes, from *S. pombe* or *S. japonicus* total polar lipid fractions (TPLs). In addition to GPLs, TPLs contained ∼3%–5% sphingolipids (mainly ceramides). These GUVs were then subjected to osmotic stress to induce membrane fluctuations (Figure 2A). Analysis of thermal membrane fluctuations demonstrated that the bending rigidity parameter of membranes assembled from the *S. japonicus* TPL mixture was approximately 2-fold higher than that originating from *S. pombe* GUVs (Figure 2A). This indicated that GPL asymmetry and/or increased FA saturation in *S. japonicus* might contribute to higher bilayer stiffness.

Lipid packing is another important biophysical parameter that determines membrane properties [18]. We therefore examined the lipid lateral segregation in the bilayer of *S. pombe* and *S. japonicus* GUVs by examining the fluorescence intensity of the lipid tracer Fast DiO, which has a strong preference for the liquid disordered (Ld) over the liquid ordered (Lo) phase [19]. Our analyses revealed that GUVs of *S. pombe* at 24°C were predominantly in the Ld phase with some GUVs showing coexistence of Ld and Lo phases (Figures 2B and S2). The proportion of the Lo phase decreased with raise in temperature, with most *S. pombe* membranes becoming fully disordered at 37°C. In contrast, GUVs assembled from *S. japonicus* TPLs exhibited pronounced membrane order at 24°C. Increasing temperature resulted in the growth of Ld domains in *S. japonicus*-derived membranes but phase coexistence was maintained at 37°C, which is at the higher end of the physiologically relevant temperature range (Figure 2B). This was in spite of the lower sphingolipid to GPL ratio in this organism, as compared to *S. pombe* (Figure 1C). Taken together, these results demonstrate that the divergence in GPL structures between the two fission yeasts may have a profound effect on the biophysical properties of their membranes, resulting in higher membrane stiffness and a distinct phase behavior in *S. japonicus*.

### Cytosolic Fatty Acid Synthase Drives C10:0 Synthesis in *S. japonicus*

**Figure 3:**
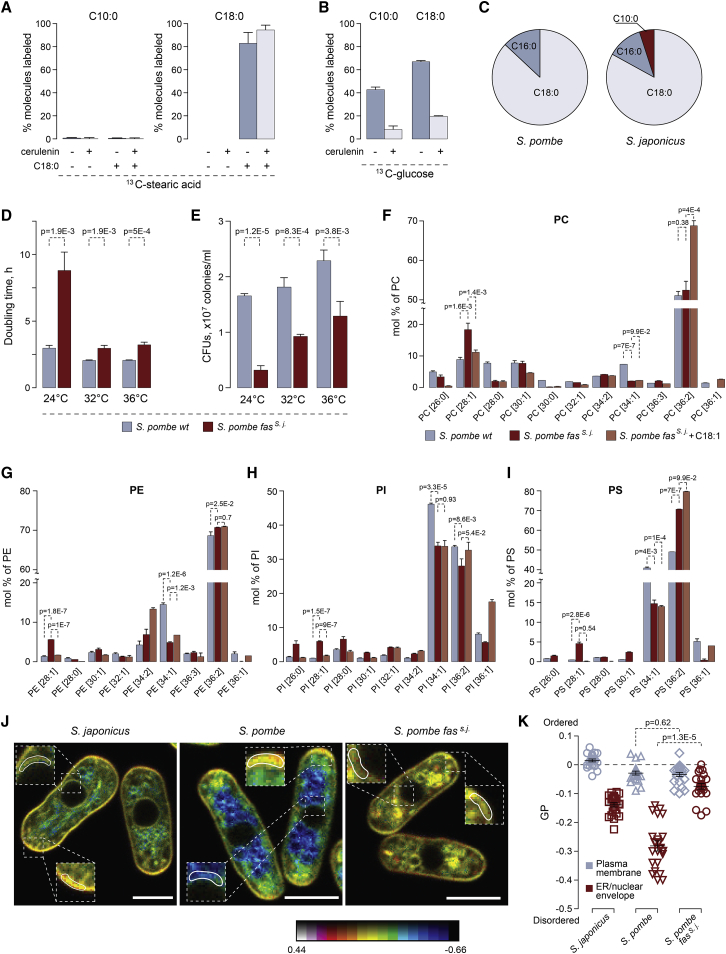
C10:0 Is Synthesized in Cytosol by the FAS Complex (A) GC-MS analysis of ^13^C-labeled C10:0 and C18:0 FAs extracted from *S. japonicus* grown in the presence of the U-^13^C-C18:0 for 4 h. (B) GC-MS analysis of ^13^C-labeled C10:0 and C18:0 FAs extracted from *S. japonicus* grown in the presence of the U-^13^C-glucose for 4 h. (C) A pie chart summarizing the results of shotgun mass spectrometry analysis of FA-CoA products generated *in vitro* by purified cytosolic FA synthases from *S. pombe* and *S. japonicus*. Shown are percentages of acyl-CoA species identified in each reaction. (D) Doubling times for *S. pombe* wild-type and *fas*^*s. j.*^ cultures grown at indicated temperatures in the yeast extract with supplements (YES) medium (n = *3*). (E) Survival of the *S. pombe* wild-type and *fas*^*s. j.*^ cells at indicated temperatures, in CFUs per mL of culture. (F–I) Graphs representing molecular species profiles for PC (F), PE (G), PI (H), and PS (I) in *S. pombe* wild-type, *fas*^*s. j*^· and *fas*^*s. j*^· cells grown in the presence of C18:1. Note a frequent increase in 28:1 at the expense of 34:1 species. Shown are the mean values ± SD (n = 3). p values are derived from the unpaired parametric t test. (J) Single-plane pseudocolored generalized polarization (GP) images. Color bar designates the range of GP values where reds show high membrane order and blues show low membrane order. Zoomed regions indicate plasma membrane and NE/ER zones used to quantify mean GP values. Scale bar, 5 μm. (K) A plot representing mean GP values quantified at the plasma membrane region at the tip of the cell and at the NE/ER region in cells of indicated genotypes (22 cells each). For (A) and (B), 10 μM cerulenin was added when indicated. Shown are the means of percentages of ^13^C-labeled FAs ± SD (n = 6). See also Figure S3 and Table S2.

We then set out to understand biosynthetic origins of the C10:0 abundance in *S. japonicus*. Using metabolic labeling followed by gas chromatography-mass spectrometry (GC-MS), we ruled out the possible shortening of LCFAs as a source of this medium-chain fatty acid (MCFA). Inhibition of endogenous FA synthesis by cerulenin and subsequent supplementation with stably labeled stearic acid (^13^C-C18:0) did not result in the detection of ^13^C-labeled C10:0 (Figure 3A), which was in line with our observation that supplementation with LCFAs did not rescue *S. japonicus* growth upon fatty acid synthase (FAS) inhibition (Figures 1K–1L). This suggested that C10:0 likely originated through *de novo* FA synthesis. Indeed, analysis *of S. japonicus* cells grown in the presence of ^13^C-labeled glucose revealed that the rates of synthesis were comparable between C10:0 and other FAs and that all FA production was inhibited by the FAS inhibitor cerulenin (Figures 3B and S3A).

Fungi and animals encode two pathways for *de novo* FA biosynthesis—located in the mitochondria or the cytosol. Deletion of three genes (*htd2Δ*, *mct1Δ*, and *etr1Δ*), whose products are predicted to execute essential steps of mitochondrial FA synthesis [20], did not result in changes to the relative abundance of C10:0 in *S. japonicus* (Figure S3B). The cytosolic fatty acid synthase (FAS) is a multi-enzyme complex containing all catalytic activities required for the reaction sequence as discrete functional domains [21]. We purified FAS complexes from either *S. pombe* or *S. japonicus* and reconstituted FA synthesis *in vitro*. The FAS activity was monitored by measuring the malonyl-CoA- and acetyl-CoA-dependent rates of NADPH oxidation (Figure S3C; [22]). Mass spectrometry analyses of the reaction products revealed that, in contrast to the *S. pombe* FAS that produced only C18:0 and some C16:0, the purified *S. japonicus* enzyme was able to synthesize C10:0 alongside the LCFAs, albeit with a lower yield compared to the steady-state abundance of C10:0 *in vivo* (Figure 3C; Table S2).

We wondered whether the genetic exchange of the *S. pombe* cytosolic FAS to its *S. japonicus* counterpart would be sufficient to alter the spectrum of FA products and, as a result, structural composition of GPLs. The fission yeast FAS is composed of α and β subunits forming an α_6_β_6_ oligomer [21]. We substituted the open reading frames (ORFs) encoding both the α and the β subunits (*fas2* and *fas1*, respectively) in *S. pombe* with their *S. japonicus* orthologs, maintaining the native regulatory contexts unchanged. Interestingly, replacement of the *S. pombe* FAS with the corresponding complex from *S. japonicus* (*fas*^*s. j*^*^.^*) led to cells dividing at smaller size (Figure S3D), increased population doubling times (Figure 3D), and decreased cell survival (Figure 3E) across the physiological range of temperatures. The growth defect was particularly pronounced at 24°C in both rich and minimal media (Figures 3D and S3E). *S. pombe fas*^*s. j*^*^.^* cultures grew at comparable rates in glucose-rich and respiratory media (Figure S3F) and consumed oxygen similarly to the wild type (Figure S3G), indicating that mitochondrial function was not overtly affected by FAS substitution. Addition of exogenous oleic acid (C18:1) to *S. pombe fas*^*s. j*^*^.^* cultures rescued their doubling times, which suggested that the enhanced C10:0 production could be at root of slow growth (Figure S3H).

Indeed, we observed an increase in lower molecular weight species (mostly C28:1) for all major GPL classes in the engineered *S. pombe* strain (Figures 3F–3I and S3I; Table 6 in Data S1). Fragmentation analyses showed that such lipids contained C18 FAs in the *sn-1* and C10:0 in the *sn-2* positions of the glycerol backbone (as an example, see MS/MS spectra for PI C28:1 in Figure S3J. Interestingly, an increase in the C28:1 species in most classes coincided with decreased abundance of C34:1 lipids containing C18:1 and C16:0 chains (Figures 3F–3I and S3I). This suggested that the *S. japonicus* FAS was sufficient to increase C10:0 content at the expense of C16:0 and that C10:0 was incorporated in place of a saturated long FA chain into *S. pombe* GPLs. Additionally, we detected increased levels of ceramide, phosphatidic acid (PA), sterols, and a slightly increased PC/PE ratio in *S. pombe* expressing the transplanted *S. japonicus* FAS (Figures S3K and S3L; Table 6 in Data S1). As expected from its rescue of growth and survival, oleic acid treatment led to the decrease in C10:0-containing GPL species in *S. pombe fas*^*s. j*^*^.^* cells (Figures 3F–3I and S3I). It rescued changes in the abundance of some but not all lipid classes and induced further disbalance in the PC/PE ratio (Figure S3K and S3L).

We explored potential changes in subcellular membrane organization caused by the transplantation of *S. japonicus* FAS to *S. pombe* using the ratiometric dye di-4-ANEPPDHQ [23]. Curiously, the NE-endoplasmic reticulum (ER) membranes became more ordered in *S. pombe fas*^*s. j*^*^.^* cells, whereas the plasma membrane lipid order did not change. This observation was broadly in line with the measurements of *S. japonicus*, which revealed a considerably higher NE/ER lipid order as compared to the wild-type *S. pombe* (Figures 3J and 3K).

Although *S. japonicus* FAS was sufficient to synthesize C10:0 both *in vitro* and *in vivo*, the relative amounts of C10:0 were lower compared to *S. japonicus* cells. This suggests that other factors may enhance the efficiency of C10:0 synthesis in that organism. The acyl-CoA-binding protein (ACBP) was shown to be involved in FA termination and transport and, when in excess, to shift the FAS product spectrum toward MCFAs *in vitro* [24]. However, neither deletion nor replacement of the gene encoding the *S. japonicus* ACBP (*acb1*) with its *S. pombe* ortholog led to significant changes in the relative amount of C10:0 as compared to the wild-type *S. japonicus* (Figure S3M). This indicated that the generation of C10:0 in *S. japonicus* does not rely on ACBP activity, and other factors will need to be examined in future studies.

### Replacement of the *S. pombe* Fatty Acid Synthase with Its *S. japonicus* Counterpart Causes UPR Activation and Affects the Secretory Pathway

**Figure 4:**
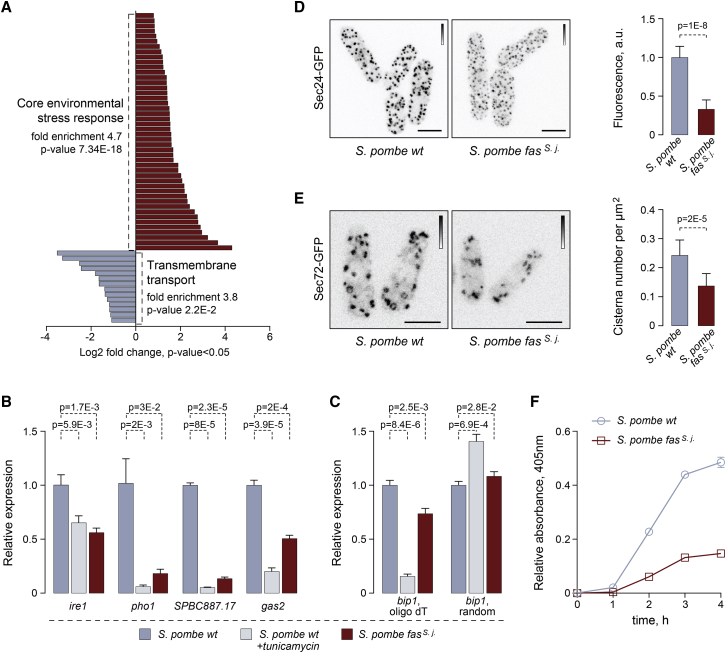
Genetic Replacement of the Cytosolic FAS Complex in *S. pombe* with Its S. *japonicus* Counterpart Results in UPR Activation and Defects in Secretory Pathway (A) Differentially expressed genes constituting two largest functional categories in *S. pombe fas*^*s. j.*^ cells as compared to the wild type. Cells were grown in YES at 24°C. AnGeLi suite was used for GO annotations. (B) qPCR analyses of indicated poly(A) mRNAs in *S. pombe* wild type and wild-type treated with 0.5 μg/mL tunicamycin for 1 h to induce the UPR and *fas*^*s. j.*^ mutant cells. (C) qPCR analyses of *bip1* poly(A) and total mRNA species in *S. pombe* wild type and wild-type treated with 0.5 μg/mL tunicamycin for 1 h and *fas*^*s. j.*^ mutant cells. (D) Maximum intensity z-projections of spinning-disk confocal images of *S. pombe* wild-type and *fas*^*s. j.*^ cells expressing Sec24-GFP. Average fluorescence intensities relative to the wild type are presented on the right. n = 142 and 154 cells for the wild type and the mutant, respectively. (E) Maximum intensity z-projections of spinning-disk confocal images of *S. pombe* wild-type and *fas*^*s. j.*^ cells expressing Sec72-GFP. For each genotype, *trans*-Golgi cisternae numbers per unit of area are presented on the right. n = 153 and 167 cells for the wild-type and the mutant, respectively. (F) The efficiency of acid phosphatase secretion measured in *S. pombe* wild-type and *fas*^*s. j.*^ cells. Shown are the mean values ± SD (n = 3). Shown in (B) and (C) are the mean values derived from three biological and two technical repeats, normalized to the wild type. p values are derived from the unpaired parametric t test. In (D) and (E), scale bars represent 5 μm. Calibration bars are shown for each image. See also Figure S4.

*S. pombe fas*^*s. j*^*^.^* cells exhibited global changes in mRNA abundance as compared to the wild type, as assessed by RNA sequencing (RNA-seq), both at 24°C and 30°C. Differentially expressed genes were broadly comparable between the two growth conditions, although we also detected some potential temperature-specific regulation events (Figure S4A; Tables vii and viii in Data S1). The largest group of upregulated transcripts included the core environmental stress-response (CESR) genes (Figures 4A and S4B; Tables vii and viii in Data S1). Yet another group of upregulated mRNAs including FA metabolism genes was previously identified as targets of the transcriptional regulator of lipid homeostasis Mga2 [25] (9.88-fold enrichment, p = 1.94E-05; Table 7 in Data S1).

Of note, we observed a striking enrichment of the gene ontology (GO) term “transmembrane transport” among significantly downregulated genes (Figures 4A and S4B; Tables vii and viii in Data S1). This suggested a possibility of a global membrane-related cellular response to the FAS substitution. Indeed, several downregulated genes, including *pho1* encoding a major secreted acid phosphatase [26], were previously identified as targets of the RIDD-type unfolded protein response (UPR) in *S. pombe*. In this organism, a transmembrane ER-resident kinase/endonuclease Ire1 governs the decay of ER-associated mRNAs, working together with the downstream mRNA no-go-decay machinery [27, 28]. An RT-qPCR-based expression analysis of a number of Ire1 targets, including those identified in our RNA-seq datasets, revealed a significant decrease in their steady-state mRNA levels in *S. pombe fas*^*s. j*^*^.^* cells as compared to the wild type (Figures 4B and S4C).

As expected, similar, albeit more pronounced downregulation was observed in cells treated with the inducer of ER stress, tunicamycin (Figures 4B and S4C). Unlike other UPR targets, the *bip1* mRNA is stabilized by the cleavage of its poly(A) tail, supporting activity of this major chaperone upon ER stress [27]. In line with this, we observed a decline in poly(A) *bip1* mRNA but an overall message stabilization in *S. pombe fas*^*s. j*^*^.^* mutants (Figure 4C). Importantly, *ire1* deletion was synthetically lethal with *fas*^*s. j*^*^.^* replacement. Together, these data are consistent with the hypothesis that substituting the *S. pombe* FAS with the *S. japonicus* complex leads to a chronic activation of the UPR and that survival of the retroengineered cells relies on the presence of this ER homeostasis pathway.

We reasoned that the presence of the unusual C10:0-containing asymmetrical membrane GPLs and increase in ER membrane order in *S. pombe fas*^*s. j*^*^.^* cells may impact on the organization and function of the secretory pathway, where lipid composition is tightly regulated [29]. Indeed, we observed a drastic decrease in the fluorescence intensity of the transitional ER marker Sec24-GFP [30] (Figures 4D and S4D). Furthermore, the *fas*^*s. j*^*^.^* mutant cells exhibited a reduced number of *trans*-Golgi cisterna (Figure 4E). Consistent with UPR-dependent downregulation of *pho1* mRNA (Figure 3D) and general defects in ER and Golgi organization, *S. pombe fas*^*s. j*^*^.^* cells were severely defective in acid phosphatase secretion (Figure 4F). Thus, introduction of asymmetrical phospholipids to *S. pombe* leads to pronounced defects in functions of the secretory pathway.

### Evidence for Evolutionary Adaptation of Transmembrane Domains to Membrane Lipid Composition

**Figure 5:**
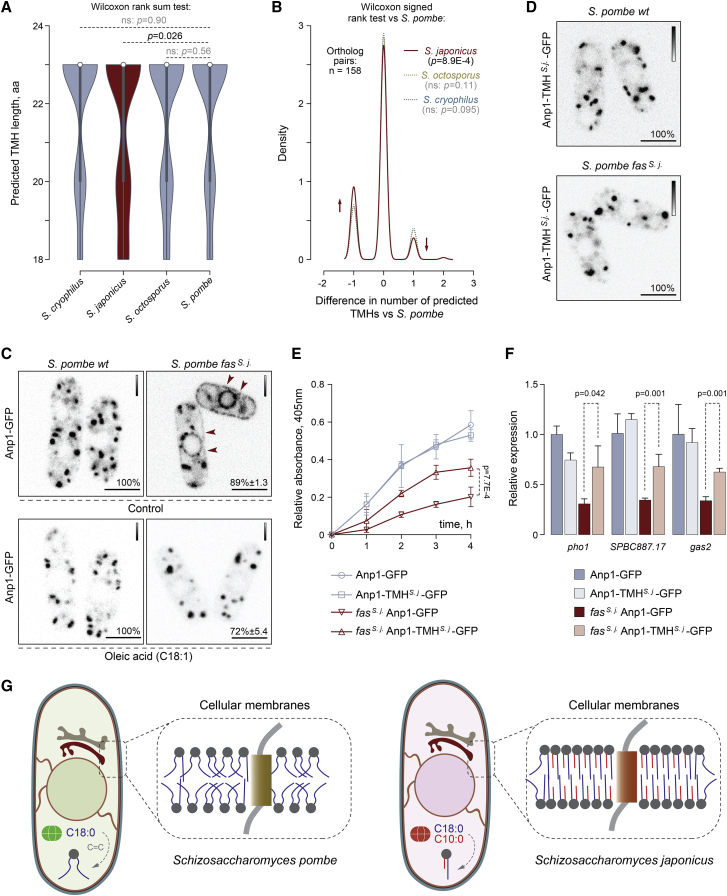
Transmembrane Helices May Have Co-evolved with Membrane Lipids in *S. japonicus* (A) A violin plot for distributions of predicted TMH lengths in the four fission yeast species. 1:1:1:1 orthologs of single-span TMH proteins were selected for the analysis. p values are derived from the Wilcoxon rank-sum test. (B) A density trace graph summarizing pairwise differences in the number of predicted TMHs in *S. japonicus*, *S. octosporus*, and *S. cryophilus*, as compared to *S. pombe*. Only 1:1:1:1 orthologs of *S. pombe* single-span TMH proteins were included. p values are derived from the Wilcoxon rank-sum test. (C) Single-plane spinning-disk confocal images of cells expressing Anp1-GFP in *S. pombe* wild-type and *fas*^*s. j.*^ cells in the absence (n = 205 and n = 153 cells, respectively) or the presence of C18:1 supplementation (n = 109 and n = 97 cells, respectively). (D) Single-plane spinning-disk confocal images of cells expressing Anp1-TMH^S.j.^-GFP as a sole copy of the protein (107 cells with endogenous FAS and 110 cells with replaced FAS were counted). (E) A graph summarizing the results of the acid phosphatase secretion assay in *S. pombe* cultures of indicated genotypes. Shown are the mean values ± SD (n = 3). (F) A graph summarizing the results of qPCR analyses of indicated poly(A) mRNAs. Shown are the mean values derived from three biological and two technical repeats, normalized to the wild type. p values are derived from the unpaired parametric t test. (G) A diagram summarizing our hypothesis on co-evolution of transmembrane helices and membrane lipids in *S. pombe* and *S. japonicus*. In (C) and (D), scale bars represent 5 μm. Calibration bars are shown for each image. Included are the percentages of cells in a population exhibiting the indicated phenotypes. In (E) and (F), p values are derived from the unpaired parametric t test. See also Figure S5.

Membrane composition and physical properties may impose constraints on the structure of transmembrane parts of integral membrane proteins [31, 32, 33]. The comparisons of predicted transmembrane helices (TMHs) in all orthologous groups of transmembrane proteins between *S. pombe* and other fission yeast species (1:1:1:1 orthologs) showed that predicted *S. japonicus* TMHs contained many small non-polar amino acids and fewer large non-polar residues compared to the rest of fission yeasts (Figures S5A–S5C). Interestingly, the pairwise comparison of predicted TMHs in single-TMH proteins revealed a statistically significant enrichment of relatively short TMHs in *S. japonicus* (Figure 5A; Table 9 in Data S1). For a number of *S. japonicus* proteins, TMHs failed to be predicted with confidence altogether (Figure 5B; Table 9 in Data S1). Thus, it appears that transmembrane domains of at least some orthologous proteins in *S. japonicus* diverged greatly compared to those in other fission yeasts. Such differences might be a result of structural adaptation of TMHs in *S. japonicus* to profound changes in the FA composition of membrane phospholipids.

We reasoned that *S. pombe* proteins, orthologs of which exhibit TMH shortening or poor TMH prediction in *S. japonicus* as compared to other fission yeast species, might be particularly vulnerable to the introduction of the *S. japonicus* FAS to *S. pombe* and, hence, an increase in GPL acyl chain asymmetry. To test this hypothesis, we visualized the subcellular localization of four TMH-divergent candidates, predicted to localize to various membrane sub-compartments. A mannosyltransferase complex subunit Anp1 that normally localizes to *cis*-Golgi [30] was mis-targeted in *S. pombe fas*^*s. j*^*^.^* cells, partitioning clearly between the ER and Golgi (Figure 5C). Correction of cellular FA composition by exogenous oleic acid (C18:1) led to the reversal of this mis-localization pattern (Figure 5C). The mis-localization was not due to UPR engagement as Anp1-GFP did not relocalize to the ER in tunicamycin-treated cells (Figure S5D). The Anp1-interacting protein Gmh5/Mnn10 [34] was also retained in the ER in *S. pombe fas*^*s. j*^*^.^* cells (Figure S5E), suggesting that the localization and function of this entire complex could be affected. Curiously, the hydrophobic helix in *S. japonicus* Gmh5 does not reach the TMH prediction threshold, unlike its *S. pombe* counterpart (Table 9 in Data S1). Mis-targeting phenotypes were observed for two other protein candidates that were predicted to have a shorter TMH or failed to reach the TMH prediction cutoffs in *S. japonicus*. The ER-lipid droplet diacylglycerol transferase Lro1 [35] became strongly enriched at the perinuclear ER while the fluorescence intensity of the GFP-tagged ER-plasma membrane tethering protein Tcb2 increased and it was now detected throughout the entire ER network [36] (Figure S5E).

The predicted TMH in Anp1 was 18 amino acids in *S. japonicus* as compared to 20 amino acids in *S. pombe* and 23 amino acids in other fission yeasts (Figure S5F). Despite this difference, endogenous Anp1 proteins exhibited a typical Golgi localization in both *S. pombe* and *S. japonicus* (Figures 5C and S5G). To establish whether mis-localization of Anp1 in *S. pombe fas*^*s. j*^*^.^* cells could be driven by a mismatch between its TMH structure and the rewired membrane lipid composition, we engineered an Anp1 chimeras in *S. pombe* by replacing the native TMH with its shorter *S. japonicus* version and keeping the rest of the protein intact (Figure S5F). Strikingly, this was sufficient to restore the Golgi localization pattern of Anp1 in *S. pombe fas*^*s. j*^· cells (Figure 5D). The swap did not affect Anp1 Golgi localization in the wild type, indicating that the *S. japonicus* TMH is compatible with both types of membranes.

We did not observe significant differences in the growth rates between the Anp1-GFP and the Anp1-TMH^*S. j.-*^GFP *S. pombe fas*^*s. j*^*^.^* cultures (Figure S5H). Yet, recovering Anp1 Golgi localization by the TMH swap led to a significant improvement in acid phosphatase secretion efficiency in *S. pombe fas*^*s. j*^*^.^* cells (Figure 5E). Since *pho1* is a target of the UPR pathway (Figure 4B), we tested whether this could be due to a higher abundance of the *pho1* mRNA. Indeed, the steady-state levels of *pho1* poly(A) mRNA and other UPR targets were partially rescued in *S. pombe fas*^*s. j*^*^.^* cells expressing Anp1-TMH^*S. j.-*^GFP (Figure 5F). Thus, restoring the localization of the Anp1-containing mannosyltransferase complex to Golgi is sufficient to ameliorate at least some of the defects induced by grafting the *S. japonicus* FAS to *S. pombe*.

## Discussion

We show that *S. japonicus*, a sister species to a well-studied model organism *S. pombe*, synthesizes abundant structurally asymmetrical phospholipids, where the medium acyl chain C10:0 is linked to the *sn*-2 position of the glycerol backbone and the *sn*-1 position is occupied by a long, usually saturated FA (Figure 1). *In vitro*, membranes assembled from *S. japonicus* TPL mixtures are more rigid and ordered (Figure 2) [37]. The FA tails of *S. japonicus* phospholipids are considerably more saturated (Figure 1E) and therefore are likely to form closely packed and more ordered bilayers. A large difference in the lengths of two acyl chains of individual molecules may result in partial interdigitation of FAs, also leading to an increase in the strength of lateral interactions and tighter packing [38, 39], although interdigitation is likely dynamic in liquid phase [40, 41]. *In vivo*, membrane biophysical properties may be further modified by sterols and sphingolipids. Of note, in spite of being more ordered, *S. japonicus* membranes have a lower sphingolipid/GPL ratio as compared to *S. pombe* (Figure 1C). The caveat of *in vitro* experiments using TPL fractions is that the different abundance of subcellular membrane compartments might contribute to the observed differences in membrane biophysics. However, *S. japonicus* endomembranes are also more ordered *in vivo* (Figures 3J and 3K), suggesting that their unique GPL architecture might have a major impact on membrane properties. Further work will be required to assess how asymmetrical lipids are partitioned within the cell and the bilayer and how this affects cellular physiology.

The molecular changes underlying production of large amounts of C10:0 alongside the LCFAs and assembly of asymmetrical phospholipids might have allowed *S. japonicus* to colonize new ecological niches. Both FA desaturation and sterol biosynthesis—normally required for maintaining membrane fluidity—are oxygen-demanding processes [42, 43]. Curiously, anaerobically grown budding yeast synthesize asymmetrical MCFA-containing GPLs [44]. Similarly, defective desaturation upregulates the synthesis of MCFAs even in aerobic conditions [45]. This suggests that phospholipid asymmetry might provide an alternative way of maintaining membrane physico-chemical properties when FA desaturation and/or sterol synthesis is not possible. In line with possible adaptation to anaerobic lifestyle, *S. japonicus* does not respire, grows well in the absence of oxygen, and undergoes robust transitions between yeast and burrowing hyphae [46, 47, 48, 49]. Phospholipid asymmetry and FA tail saturation both reduce membrane permeability to water, small solutes, and/or oxygen [50, 51, 52, 53], which might provide additional advantages in the wild. Budding yeast and *S. pombe* utilize specialized gene expression programs executed by the membrane-sensing transcription factor Mga2 [54] to sustain FA desaturation in low-oxygen conditions [25, 55]. In the future, it will be of interest to assess the wiring of this network in *S. japonicus*, given a markedly distinct physiology of the organism.

*S. japonicus* FAS exhibits overall sequence conservation with other fungal FA synthases (see STAR Methods for corrected *fas1* ORF), although it does have a number of potentially interesting substitutions to be analyzed in future. The budding yeast mutagenesis experiments suggest that the evolutionary acquisition of new FAS functionality may not necessarily require a large number of steps [56]. Importantly, other cellular factors may influence the spectrum of FAS products. An interesting possibility could be the different ratios between acetyl- and malonyl-CoA in *S. japonicus* and *S. pombe*. Relative depletion of malonyl-CoA results in the enzyme favoring priming over elongation [56] and increases the production of shorter FAs, whereas hyperactivation of the enzyme responsible for malonyl-CoA synthesis shifts FA distribution toward longer lengths [57]. Curiously, the mRNA for this enzyme, *cut6* [58], is significantly upregulated in *S. pombe* cells expressing *S. japonicus* FAS (Tables vii and viii in Data S1), perhaps as a part of a Mga2-based compensation mechanism triggered by elevated C10:0 levels. Other cellular adaptations are likely required to efficiently handle C10:0 alongside LCFAs, including modifications of lipid remodeling enzymes.

Our bioinformatics analyzes indicate that many *S. japonicus* transmembrane proteins might have experienced the need to adapt to changes in cellular lipid composition by reducing an average residue volume or shortening and/or remodeling the TM domains (Figures 5A, 5B, and S5A–S5C). TMDs span the hydrophobic core of the lipid bilayer, and therefore specific membrane FA composition may impose constraints on their sequences [32, 33]. TMDs of single-spanning ER and *cis*-Golgi proteins tend to be shorter than those residing in the later compartments of the secretory pathway [31]. Curiously, most single-pass TM proteins that exhibit shortened TMDs in *S. japonicus* as compared to other fission yeasts are predicted to function largely at the ER, ER-organelle contacts, and *cis*-Golgi (Table 9 in Data S1). The membranes of the broad ER territory tend to exhibit looser lipid packing [18], and thus their protein residents could be particularly sensitive to an increase in membrane order. In addition to reducing the hydrophobic length, other properties of the TMDs may facilitate their insertion and/or folding in well-ordered membranes—perhaps explaining the statistically significant increase in poorly predicted TMHs in *S. japonicus* single-pass TM proteins versus their orthologs in other fission yeasts.

Supporting the hypothesis that TMHs co-evolve with membranes [31, 32, 33], the replacement of the TMH in *S. pombe* Anp1 with a shorter *S. japonicus* version rescues its mis-targeting in *S. pombe* expressing the *S. japonicus* FAS (Figure 5D) and partially relieves downregulation of UPR targets in this genetic background (Figures 5E and 5F). Anp1 is a key player in the assembly of the mannosyltransferase complex, which takes place in the ER before trafficking to Golgi [34, 59]. It is possible that ER retention of this complex impacts folding of proteins destined for secretion through improper glycosylation events, activating UPR signaling. Yet, given the complexity of observed cellular phenotypes, the physiological consequences of introducing the *S. japonicus* FAS to *S. pombe* are likely not limited to this particular pathway. Poor growth and stress-response activation in *fas*^*s. j*^*^.^* cells (Figures 3D, 3E, 4A, S3E, and S4B) may be attributed not only to downregulation of a large number of membrane transporters but also the changes in membrane biophysical properties (Figures 3J and 3K), which could affect signaling through the cellular stress pathways [60, 61]. Ire1 may also sense bilayer stress directly [62, 63, 64]. Given the reliance of *S. japonicus* on C10:0-containing largely saturated phospholipids, it will be interesting to see whether this organism might have rearranged the structural features of Ire1 allowing it to sense ER lipid composition.

Our work provides new insights into the generation of phospholipid diversity in evolution and its impact on cellular biology connected to membrane functions. We propose that evolutionary changes in membrane FA composition may necessitate rapid adaptation of the transmembrane domains (Figure 5G). The two fission yeast species with their divergent lifestyles constitute an ideal experiment of Nature, study of which may lead to a conceptually new understanding of the relationship between the underlying metabolic makeup and the evolution of cellular properties.

## STAR★Methods

### Key Resources Table

**Table:** 

REAGENT or RESOURCE	SOURCE	IDENTIFIER
**Chemicals, Peptides, and Recombinant Proteins**		

Cerulenin	Sigma-Aldrich	C2389
DMSO	Sigma-Aldrich	D8418
C10:0 (methylated)	Sigma-Aldrich	299030
C18:0	Sigma-Aldrich	S4751
C18:1	Sigma-Aldrich	O1008
FA-free BSA	Sigma-Aldrich	A7030
Methanol for LC-MS Optima	Thermo Fisher	A456
Propan-2-ol for LC-MS Optima	Thermo Fisher	A461
Chloroform suprasolv	Merck	102432
Dimethylformamide Uvasol	Merck	102937
Ammonium chloride extra pure	Thermo Fisher	12125-02-9
Acetyl chloride	Sigma-Aldrich	00990
di20:0 PC	Larodan	37-2000-9
Cholesterol	Sigma-Aldrich	C8667
Cholesteryl oleate	Sigma-Aldrich	C9253
16:0 D31-18:1 PC	Avanti Polar Lipids	860399C
16:0 D31-18:1 PE	Avanti Polar Lipids	860374C
16:0 D31-18:1 PI	Avanti Polar Lipids	860042P
16:0 D31-18:1 PS	Avanti Polar Lipids	860403C
16:0 D31-18:1 PA	Avanti Polar Lipids	860453C
16:0 D31-18:1 PG	Avanti Polar Lipids	860384C
CL tetra14:0	Avanti Polar Lipids	750332C
CL tetra18:1	Avanti Polar Lipids	710335C
N-16:0 Phytosphingosine	Avanti Polar Lipids	860617P
di22:1 DG	Nu-Chek Prep	D-301
tri22:1 TG	Nu-Chek Prep	T-300
Phospholipase A2 (Snake venom from *Crotalus atrox*)	Serva	34736
FAST DIO	Molecular Probes	D3898
Di-4-ANEPPDHQ	Molecular Probes	D36802
C18:0; ^13^C labeled at each carbon atom	Cambridge Isotope Laboratories	CLM-6990-PK
^13^C labeled glucose	Cambridge Isotope Laboratories	CLM-1396-PK
Brij35	Sigma-Aldrich	8019620250
[1-^13^C_1_]-lauric acid	Cambridge Isotope Laboratories	CLM-1586-PK
MethPrep II	Grace	5122149
Pefabloc SC	Sigma-Aldrich	76307
Leupeptin	Sigma-Aldrich	108976
Pepstatin A	Sigma-Aldrich	P5318
Malonyl-CoA	Sigma-Aldrich	M4263
Acetyl-CoA	Sigma-Aldrich	A2181
NADPH	Roche	10107824001
13:0-CoA	Avanti Polar Lipids	870713
p-nitrophenyl phosphate	Sigma-Aldrich	4876

**Critical Commercial Assays**		

Micro BCA Protein Assay Kit	Thermo Fisher Scientific	23235
HiTrap Q HP 5 ml column	GE Healthcare	17115301
Amicon 10K ultra-filters	Merck	UFC900308
RNeasy Plus Mini Kit	QIAGEN	74134
polyA KAPA mRNA Hyper_Prep	Roche	08098115702
Transcriptor First Strand cDNA Synthesis Kit	Roche	04379012001
Probe Blue Mix Lo-ROX	qPCRBIO	PB20.21-01

**Deposited Data**		

Raw and analyzed RNaseq data	This study	NCBI GEO: GSE141579

**Experimental Models: Organisms/Strains**		

*S. pombe* wild type 972 h- prototroph	Oliferenko laboratory	SO7812
*S. pombe* f*as1 S. japonicus*:kanR *fas2 S. japonicus*:hygR ade6-216 ura4-D18 leu1-32 h-	This study	SO8482
*S. pombe fas1*^*S. japonicus*^*:kanR fas2*^*S. japonicus*^*:hygR* prototroph h+	This study	SO8487
*S. pombe anp1-GFP::ura4+ ade6-216 ura4-D18 leu1-32* h+	Oliferenko laboratory	SO1511
*S. pombe fas1*^*S. japonicus*^*:kanR fas2*^*S. japonicus*^*:hygR sec24-GFP:ura4+ ade6-216 ura4-D18 leu1-32*	This study	SO8496
*S. pombe sec24-GFP::ura4+ ade6-216 ura4-D18 leu1-32 h+*	Oliferenko laboratory	SO1509
*S. pombe fas1*^*S. japonicus*^*:kanR fas2*^*S. japonicus*^*:hygR sec72-GFP:ura4+ ade6-216 ura4-D18 leu1-32*	This study	SO8502
*S. pombe sec72-GFP::ura4+ ade6-216 ura4-D18 leu1-32 h+*	Oliferenko laboratory	SO1517
*S. pombe ire1Δ*	Gift from the Peter Walter laboratory	PWY1751 (yPK002) Entered to the Oliferenko collection as SO8503
*S. pombe fas1*^*S. japonicus*^*:kanR fas2*^*S. japonicus*^*:hygR lro1-GFP:leu1+ ade6-M2x ura4-D18 leu1-32*	This study	SO8515
*S. pombe lro1-GFP::leu1+ ade6-M2x ura4-D18 h+*	Oliferenko laboratory	SO7138
*S. pombe gmh5-GFP::kanR 972 h-*	This study	SO8516
*S. pombe fas1*^*S. japonicus*^*:kanR fas2*^*S. japonicus*^*:hygR gmh5-GFP::kanR*	This study	SO8517
*S. pombe tcb2-GFP::kanR 972 h-*	This study	SO8518
*S. pombe fas1*^*S. japonicus*^*:kanR fas2*^*S. japonicus*^*:hygR tcb2-GFP::kanR*	This study	SO8519
*S. pombe anp1-TMH*^*S.japonicus*^*-GFP::ura4+ ade6-210 ura4-D18 leu1-32 h-*	This study	SO8522
*S. pombe fas1*^*S. japonicus*^*:kanR fas2*^*S. japonicus*^*:hygR anp1-TMH*^*S.japonicus*^*-GFP::ura4+ ade6-210 ura4-D18 leu1-32 h-*	This study	SO8523
*S. japonicus matsj-2025 h+ (prototroph)*	Gift from the Hironori Niki laboratory	*NIG2025* Entered to the Oliferenko collection as SOJ4
*S. japonicus htd2Δ:kanR ura4sj-D3 h-*	This study	SOJ3168
*S. japonicus mct1Δ:kanR ura4sj-D3 h-*	This study	SOJ3171
*S. japonicus etr1Δ:kanR ura4sj-D3 h-*	This study	SOJ3173
*S. japonicus acb1Δ:kanR ura4sj-D3 h-*	This study	SOJ2925
*S. japonicus acb1*^*S. pombe*^*:kanR ura4sj-D3 h-*	This study	SOJ3156
*S. japonicus Anp1-GFP::ura4+ ura4sj-D3 h+*	This study	SOJ4265

**Oligonucleotides**		

All oligonucleotides are listed in Table S3	This study	

**Recombinant DNA**		

pSO820 3′UTR-5′UTR FASI S.p-ORF FASI s.j.	Genewiz	Custom synthesis
pSO918 3′UTR-5′UTR FASII S.p-ORF FASII s.j.	Genewiz	Custom synthesis
pUC57-Kan-3′UTR-5′UTR-Anp1S.p.-ORF Anp1-TMHsj	Genewiz	Custom synthesis

**Software and Algorithms**		

LipidXplorer	[65]	https://lifs.isas.de/wiki/index.php/Main_Page
MassHunter software (version B.07.02.1938)	Agilent	N/A
MANIC software, an in house-developed adaptation of the GAVIN package	[66]	N/A
Fiji	[67]	https://imagej.net/Fiji
OpenCFU	[68]	http://opencfu.sourceforge.net/
Galaxy pipeline	Open source	https://usegalaxy.org
AnGeLi	[69]	http://bahlerweb.cs.ucl.ac.uk/cgi-bin/GLA/GLA_input
Primer3	[70]	http://primer3.ut.ee/
OMA	[71]	https://omabrowser.org/oma/home/
TMHMM 2.0	[72]	http://www.cbs.dtu.dk/services/TMHMM/
ProtParam	[73]	https://web.expasy.org/protparam/
MAFFT	[74]	https://mafft.cbrc.jp/alignment/software/
R	[75]	https://www.r-project.org/
Prism 7	GraphPad Software	N/A

### Lead Contact and Materials Availability

Further information and requests for resources and reagents should be directed to and will be fulfilled by the Lead Contact, Snezhana Oliferenko (snezhka.oliferenko@crick.ac.uk). All unique/stable reagents generated in this study are available from the Lead Contact without restriction.

### Experimental Model and Subject Details

*S. pombe* and *S. japonicus* strains used in this study are listed in Key Resources Table. We used standard fission yeast media and methods [76, 77, 78]. Cells were grown in minimal EMM or rich YES media at 24°C, 30°C, 32°C or 36°C, as indicated. Temperature-controlled 200 rpm shaking incubators we used for liquid cultures. Typically, cells were pre-cultured overnight in appropriate liquid media and then sub-cultured again to reach mid-exponential phase. Mating of *S. pombe* strains was performed on YPD solid medium and *S. japonicus*, on SPA solid medium. Spores were dissected and germinated on YES agar plates.

### Method Details

#### Molecular genetics and strain husbandry

All primers are shown in Table S3. Molecular genetics manipulations were performed using PCR- or plasmid-based homologous recombination using plasmids carrying *S. japonicus ura4*, kanR, hygR or natR cloned into the pJK210-backbone (pSO550, pSO820 and pSO893, respectively). Mitochondrial FA synthesis mutants *etr1Δ*, *mct1Δ* and *htd2Δ* of *S. japonicus* were generated using standard PCR-based recombination with KanMX6 as a selection marker. To generate a parental *S. pombe* strain carrying replacement of the two-component FAS complex with its *S. japonicus* counterpart we first replaced the *fas1* open reading frame (ORF) by homologous recombination, using a construct containing the 5′- and 3′-UTRs of f*as1*^*S.pombe*^ that flanked the *fas1 ORF*^*S.japonicus*^ and the kanMX6 cassette. Using similar strategy and the construct carrying the hygR cassette as a selection marker, we then replaced the *fas2* ORF. Of note, we have identified several mis-annotations in the *S. japonicus* gene encoding the FAS β subunit (SJAG_04107). Using both RNaseq / *de novo* transcript assembly and sequencing of cDNA obtained from vegetatively grown cells we identified the following errors: 1) T at position 1240 is absent; 2) an additional C is present at position 2158; 3) both annotated introns are in fact retained. The corrected sequence will be uploaded to EnsemblFungi. Replacements of the TMH in *S. pombe* Anp1 with its *S. japonicus* version were constructed similarly. All constructs were verified by Sanger sequencing. A PCR-based method [79] was used to tag Anp1, Gmh5, Lro1 and Tcb2 at the C terminus, typically using KanMX6 or natR as a selection marker.

#### Cell growth and survival measurements

For growth rate measurements, cells were grown in EMM (unless otherwise stated) overnight, diluted to OD_595_ 0.2 and supplemented with cerulenin (10 μg/ml in DMSO) and/or FAs conjugated with BSA based on recommendations described in [80]. FA stock (5 mM) was prepared in 50% ethanol heated to 70°C. The FA solution was then conjugated with FA-free BSA at a 5:1 ratio at 37°C. Decanoic acid was used in methylated form. Final FA concentration added to the cell culture was 250μM, mixtures of FAs were added as separate stocks to the final concentration of 250μM. Control cells were treated with BSA solution. For survival measurements cells were grown in indicated conditions for 24 hours followed by serial dilutions and plating in triplicates on YES solid medium. The colony numbers were calculated after 48 hours of growth using OpenCFU software [68].

#### ESI-MS-based lipidomic analysis

Lipid standards were from Avanti Polar Lipids (Alabaster, AL, USA), Larodan (Solna, Sweden), Nu-Chek Prep (Elysian, MN, USA), and Sigma-Aldrich (Steinheim, Germany). Solvents for extraction and MS analyses were liquid chromatographic grade (Merck, Darmstadt, Germany) and Optima LC-MS grade (Thermo Fisher Scientific, Waltham, MA, USA). All other chemicals were the best available grade purchased from Sigma-Aldrich or Thermo Fisher Scientific. Phospholipase A2 (Snake venom from *Crotalus atrox*) was from Serva (Heidelberg, Germany).

Exponentially growing yeast cell cultures in EMM were harvested and disrupted in water using a bullet blender homogenizer (Bullet Blender Gold, Next Advance, Inc., Averill Park, NY, USA) in the presence of zirconium oxide beads (0.5 mm) at speed 8 for 3 min at 4°C. Protein concentration of cell homogenates was determined using the Micro BCA Protein Assay Kit (Thermo Fisher Scientific, Waltham, MA, USA). A portion (40 μL of ∼500 μL total volume) of the homogenate was immediately subjected to a simple one-phase methanolic (MeOH) lipid extraction [11]. First, the homogenate was sonicated in 1 mL MeOH containing 2 μg di20:0 PC and 1 μg cholesterol (as extraction standards) and 0.001% butylated hydroxytoluene (as antioxidant) in a bath sonicator for 5 min, then shaken for 5 min and centrifuged at 10,000 x*g* for 5 min. The supernatant was transferred into a new Eppendorf tube and stored at −20°C until MS measurement (∼25 nmol/ml total lipid in MeOH).

Electrospray ionization mass spectrometry (ESI-MS)-based lipidomic analyses were performed on a LTQ-Orbitrap Elite instrument (Thermo Fisher Scientific) equipped with a robotic nanoflow ion source TriVersa NanoMate (Advion BioSciences, Ithaca, NY) using chips with the diameter of 5.5-μm spraying nozzles. The ion source was controlled by Chipsoft 8.3.1 software. The ionization voltages were +1.3 kV and −1.9 kV in positive and negative mode, respectively, and the back-pressure was set at 1 psi in both modes. The temperature of the ion transfer capillary was 330°C. Acquisitions were performed at the mass range of 350-1300 *m/z* at the mass resolution Rm/z 400 = 240,000.

The lipid classes phosphatidylcholine (PC), lysophosphatidylcholine (LPC), diacylglycerol (DG), triacylglycerol (TG) and ergosteryl ester (EE) were detected and quantified using the positive ion mode, while phosphatidylethanolamine (PE), phosphatidylinositol (PI), phosphatidylserine (PS), their lyso derivatives LPE, LPI, LPS, phosphatidic acid (PA), phosphatidylglycerol (PG), cardiolipin (CL), ceramide (Cer), inositolphosphoceramide (IPC) and mannosyl- inositolphosphoceramide (MIPC) were detected and quantified using the negative ion mode. Sterols were derivatized according to [81]. 500 μL methanolic extract was evaporated to dryness and 180 μL chloroform:acetyl chloride = 5:1 mixture was added to the residue to form acetate derivatives of free sterols. The reaction mixture was left for 1h at room temperature, evaporated to dryness, and reconstituted in 100 μL chloroform:methanol = 1:1. For MS analysis, 5 μL of the derivative was diluted with 45 μL infusion solvent (chloroform:methanol:isopropanol = 1:2:1, by vol.) in the presence of 100 μM sodium carbonate. Sterol acetates were detected in the positive ion mode at +1.5 kV ionization voltage at the mass resolution Rm/z 400 = 240,000. Mass spectra were analyzed in a targeted manner, i.e., we searched for ergosterol, lanosterol, zymosterol and fecosterol.

For quantification, ∼20 μL lipid extract (corresponding to 1.2 μg protein) was diluted with 280 μL infusion solvent mixture (chloroform:methanol:iso-propanol 1:2:1, by vol.) containing an internal standard mix (71 pmol PC d31-16:0/18:1, 7 pmol DG di22:1, 5 pmol TG tri22:1, 50 pmol PE d31-16:0/18:1, 43 pmol PI d31-16:0/18:1, 24 pmol PS d31-16:0/18:1, 1 pmol PG d31-16:0/18:1, 5 pmol PA d31-16:0/18:1, 1 pmol CL tetra14:0 (for *S. pombe*), 1 pmol CL tetra18:1 (for *S. japonicus*), 4 pmol Cer t18:0/16:0, and 18 pmol CE 18:1). Next, the mixture was halved, and 5% dimethylformamide (additive for the negative ion mode) or 3 mM ammonium chloride (additive for the positive ion mode) were added to the split sample halves. 10 μL solution was infused and data were acquired for 2 minutes.

MS/MS fragmentation experiments were carried out to determine FA compositions, ratio of isobaric species and FA positions. For fragmentation, HCD collision energy values were determined for PC, PE, PI and PS, and data-dependent MS/MS fragmentation experiments were performed based on mass lists from survey scans. To determine FA side chain regiochemistry, we considered the observation that losses of the neutral carboxylic acid ([M-H-RCOOH]−) or neutral ketene moieties ([M-H-RCH = C = O]−) are more abundant from the *sn-2* position than from the *sn-1* position [82]. Such fragmentation patterns were established for LCFAs and largely for pure synthetic lipids. Therefore, it was not clear if this ‘intensity rule’ could be applied to MCFAs and complex lipid mixtures where the presence of regioisomers cannot be ruled out without their pre-separation, as in shotgun MS. We established that C10:0 is exclusively esterified in the *sn-2* position by analyzing the PLA2 digestion products. Of note, the estimates of intensities of neutral carboxylic acid or ketene losses by MS/MS confirmed this result. The *sn-2*/*sn-1* intensity ratios were generally high (> 7). Therefore, it appears that the ‘intensity rule’ may hold for shorter acyl chains and the contribution of regioisomers is negligible.

Lipids were identified by LipidXplorer software [65] by matching the m/z values of their monoisotopic peaks to the corresponding elemental composition constraints. The mass tolerance was 3 ppm. Data files generated by LipidXplorer queries were further processed by custom Excel macros.

Lipid classes and species were annotated according to the classification systems for lipids [83]. For glycerolipids, the lipid class (*sn*-1/*sn*-2) format specifies the structures of the FA side chains as well as the side chain regiochemistry, e.g., PC (16:0/18:1). In sum formulas, e.g., PC (34:1), the total numbers of carbons followed by double bonds for all chains are indicated. For sphingolipids, the sum formula, e.g., Cer (44:0:4), specifies first the total number of carbons in the long chain base and FA moiety then the sum of double bonds in the long chain base and the FA moiety followed by the sum of hydroxyl groups in the long chain base and the FA moiety. Lipidomic data were expressed as mol% of polar lipids; polar lipids were calculated as the sum of GPLs and sphingolipids. Lipidomics results are presented in Data S1, tables i-vi.

We note that some minor but important lipid classes (e.g., sphingosines, sphingosine-1-phosphates, phoshoinositides), which cannot be readily assessed by shotgun MS, are missing from the current analysis.

#### Phospholipase A2 digestion

The total polar lipid fraction (TPL) was isolated from *S. japonicus* total lipid extract by thin-layer chromatography as described in [84]. The extracted lipids were separated on Kieselgel 60 silica gel TLC plates (Merck) using hexane:diethylether:acetic acid (50:50:2, by vol.); the TPL fraction was recovered from the start lane. Approximately 1 mg TPL isolate was dissolved in 250 μL diethyl ether, and then 250 μL borate buffer (0.1 M, pH 7.0), 125 μL CaCl2 (0.01 M) and 125 μL PLA2 solution (8 mg/ml PLA2 in water) were added; the mixture was stirred at room temperature in a test tube with glass stopper. After 2 h, 200 μL reaction mixture (corresponding to ∼100 μg TPL) was centrifuged at 2400 x*g*, the upper organic layer was evaporated and extracted according to a modified Folch procedure [84]. The residue was reconstituted in 200 μL water, 500 μL methanol and 250 μL chloroform were added to form one phase (chloroform:methanol:water (1:2:0.8, by vol.)). The mixture was left at room temperature for an hour with periodical vortexing, then 750 μL chloroform and 175 μL 0.2 M KCl were added for phase separation to occur (final composition of chloroform:methanol:water (2:1:0.75, by vol.)). After vortexing, the sample was centrifuged at 600 g for 10 min. The lower organic layer was evaporated under vacuum, and the residue was reconstituted in 1 mL chloroform:methanol 1:1 (by vol.). The PLA2 digest was analyzed together with the starting TPL isolate by ESI-MS as described above.

#### GUVs preparation in DexPEG hydrogel films

DexPEG hydrogel films were prepared following previously reported methods [85]. Briefly, maleimide-modified dextran (1.5% weight solution) was cross-linked by PEG dithiol at room temperature. Typically for the preparation of 5 glass substrates with DexPEG hydrogel films, maleimide-modified dextran (75 mg) (degree of substitution = 3) was dissolved in water (4.5 g) and 23.6 mg of PEG dithiol (3400 Da) in water (0.5 g) were mixed to provide a hydrogel solution. The mixture was vortexed for 1 minute and 1 mL of hydrogel solution was immediately drop-casted on thiol functionalised microscope glass slides. The DexPEG substrates were stored at room temperature for further use.

GUVs were prepared by the swelling of *S. pombe* or *S. japonicus* TPL mixtures (prepared as described above under PLA2 digestion) on DexPEG hydrogel coated glass substrates at room temperature. Briefly, the lipids were dissolved in a mixture of methanol:chloroform (3:1) to a final concentration of 50 mg/ml. 20 μL of the desired lipid solution was then drop casted on DexPEG substrates and dried under a gentle stream of nitrogen gas for 5 minutes and placed under vacuum overnight. A growth chamber was made by placing a polydimethylsiloxane (PDMS) spacer between the hybrid lipid-DexPEG hydrogel coated slide and a microscope glass slide and clamped with crocodile clips. GUVs growth was initiated by hydrating the hybrid lipid-DexPEG film with a sucrose solution (400 μl, 100 mM sucrose). The hydrated substrates were left overnight at room temperature. Dense suspensions of GUVs were collected the following day and use for phase contrast microscopy.

#### Bending rigidity measurements

GUVs suspensions (10 - 20 μl) were diluted in an observation chamber containing 400 μl of glucose solution (150 mM) for time-lapse microscopy imaging. GUVs were imaged in phase contrast mode with 1 ms exposure time and recorded for 40 s in a Nikon Eclipse TE-2000-E inverted microscope equipped with a digital high-speed camera Orca-Flash 4.0 (Hamamatsu) and a TI-DH Dia Pillar Illuminator (100W).

The bending rigidity parameter was calculated by analyzing the thermal fluctuations of GUVs from the recorded time lapsed microscopy imaging with a home-made software and methodology as published previously [17, 86]. Fluctuation analyses were performed on 4,000 contours of individual GUVs. For small deformations, the distance from the membrane edge to the center of the vesicle about the mean edge position was Fourier-transformed to give a power spectrum that was adjusted between modes 6-20. The bending rigidity parameter was extracted from the fluctuation spectrum using Helfrich equation. A detailed mathematical description of the model can be found in [87, 88].

#### Electroformation of GUVs and labeling with the FAST DIO

Chamber for GUV electroformation consisted of two conductive ITO glasses separated by a rubber spacer of 0.3 mm. Phospholipid extracts were dissolved in chloroform/methanol (2:1) and deposited on one of the ITO coated glasses. After drying the chamber was filled with 300 μl of 0.1 M Tris-HCl pH 7.5 and 200 mM sucrose. AC field of 10 Hz, 1 V was applied to the chamber. Electroformation was conducted at 40°C for 2 hours [89].

FAST DIO (Molecular Probes) was added at a concentration of 0.1 mol% to GUV mixtures followed by spinning disk confocal imaging at excitation 488 nm. Images were acquired with the same settings and intensities of the individual GUVs were analyzed at the equatorial plane.

#### Metabolic labeling

For metabolic labeling experiments cells were grown in EMM overnight, diluted to OD_595_ 0.2 and supplemented with cerulenin (10 μg/ml in DMSO) and/or 200 μM stearic acid (C18:0; ^13^C labeled at each carbon atom) in 0.5% Brij35 for 4 hours. For glucose carbon atom tracing in newly synthesized FAs, glucose in the growth media was replaced with the uniformly labeled ^13^C glucose. Following 4-hour incubation, cells were collected and subjected to lipid extraction and sample drying.

#### GC-MS

Dried samples, containing 10 nmol internal standard ([1-^13^C_1_]-lauric acid), were biphasic-partitioned using 700 μL chloroform:methanol:water (1:3:3, v/v). Data acquisition was performed using an Agilent 7890B-7000C GC-QQQ in EI mode after derivatization of dried apolar phase by the addition of 25 μL chloroform:methanol (2:1, v/v) and 5 μL MethPrep II (Grace). GC-MS parameters were as follows: carrier gas, helium; flow rate, 0.9 ml/min; column, DB-5MS (Agilent); inlet, 250°C; temperature gradient, 70°C (1 min), ramp to 230°C (15°C/min, 2 min hold), ramp to 325°C (25°C/min, 3 min hold). Scan range was m/z 50-550. Data was acquired using MassHunter software (version B.07.02.1938). Data analysis was performed using MANIC software, an in house-developed adaptation of the GAVIN package [66]. Fatty acid methyl esters (FAMES) were identified and quantified by comparison to authentic standards, and label incorporation estimated as the percentage of the metabolite pool containing one or more ^13^C atoms after correction for natural abundance.

#### FAS purification and FA synthesis *in vitro*

The procedure was adapted from [90], with some modifications. Cell were grown in YES media in a 40-l fermenter for 24 hours up to OD_595_ 0.8, collected and washed with the buffer A (50 mM Tris-HCl pH 7.5, 200 mM NaCl, 1 mM EDTA, 1 mM TCEP), followed by resuspension in the same buffer with protease inhibitors (Pefabloc SC 1 mM, leupeptin 10 μg/ml, pepstatin A 10 μg/ml). Extracts were then frozen rapidly by dripping into liquid nitrogen. Freezer mill (SPEX CertiPrep 6850 Freezer/Mill) with 7 cycles of 2 min at crushing rate of 15 was used to disrupt cells and obtain powder for protein purification. Cell powder (15 g) was resuspended in buffer A with protease inhibitors to a final volume of 150 mL and placed on ice followed by ammonium sulfate protein precipitation. First, ammonium sulfate was added slowly with vigorous mixing at 0°C to the final concentration of 23%, followed by incubation for 30 minutes. Following centrifugation at 48000 x*g* for 40 minutes pellets were discarded. Ammonium sulfate was again added to the supernatant as described above, to the final concentration of 55%. Following centrifugation as above, protein pellets were resuspended in 200 mL of buffer A and subjected to anion exchange chromatography using the HiTrap Q HP 5 mL column. Protein fractions were collected from 100 to 800 mM NaCl and analyzed for the presence of FAS activity. Typically, fractions containing active FAS eluted within the 150-300 mM NaCl range. These active fractions were combined and concentrated to 1 mL using Amicon 10K ultra-filters. Protein concentrates were then loaded on a 10%–40% linear sucrose density gradient in buffer A, prepared in 32S Beckman tubes. Those were centrifuged at 89000 x*g* for 18 hours at 4°C. Afterward, 1 mL fractions were collected from top to bottom and assayed for FAS activity. Fractions containing concentrated active enzyme (typically, three) were yellow due to FAS binding to FMN [91]. To concentrate the activity and exchange buffer systems we then used MonoQ 5/50 GL anion exchange chromatography. 500 μl MonoQ fractions were collected and verified for FAS activity. Typically 1-2 fractions exhibited high activity and those were used for *in vitro* FA synthesis.

Monitoring of NADPH oxidation by malonyl-CoA and acetyl-CoA at 340 nm was used to assess FAS activity for each step of FAS purification, as shown in [90]. The assay buffer consisted of 200 mM potassium phosphate pH 7.3, 50 μM acetyl-CoA, 25 μM malonyl-CoA, 100 μM NADPH, 2.5 mM EDTA, 4 mM dithiothreitol, and 0.03% bovine serum albumin. All components and the enzyme, except malonyl-CoA were mixed and a blank rate was measured for 20 minutes. The enzymatic reaction was then initiated by the addition of malonyl-CoA.

To analyze FAS product spectrum, active protein fractions after the MonoQ purification step were combined so that the total volume of the reaction was 400 μl. The buffered solution (200 mM potassium phosphate pH 7.3, 87.5 μM DTT, 2.25 mM NADPH, 0.20 mM acetyl-CoA, 1.00 mM malonyl-CoA) with added enzyme was kept at room temperature for 18 hours and the reactions were stopped by flash freezing.

#### Fatty acid synthase assay – product analysis

FA-CoA assay products were extracted from the reaction mixture using a previously described protocol [90] with some modifications. Briefly, 4 volumes of ice-cold acetone and 3 nmol 13:0-CoA were added to 350 μL of FAS reaction mixture. The mixture was vortexed for 30 s, transferred for 1 hour to −20°C, and then centrifuged at 10,000 x*g* for 5 min at 4°C. The supernatant was evaporated under vacuum, and the residue was dissolved in 100 μL water by sonication for 5 min. The solution was centrifuged again at 10,000 x*g* for 5 min, the supernatant was transferred into a new Eppendorf tube, and stored at −20°C until mass-spectrometry analysis (which was performed within 2 days). For mass spectrometry measurements, the assay products were first diluted 50-fold with water, then a further 12-fold dilution was made by water:acetonitrile (containing 0.1% triethylamine) 1:1 (v/v). The diluted solutions were injected into the mass spectrometer in the negative ion mode at −1.5 kV ionization voltage at the mass resolution R_m/z 400_ = 240,000. For FA-CoA detection, the MS was programmed in SIM mode with 4 Da windows for the potential FA-CoAs, while MS/MS fragmentation experiments were performed at 25% normalized HCD energy. Quantitation and product profile determination was made based on the spiked 13:0-CoA amount. To exclude the possibility of non-enzymatic product formation, blank assay mixtures (i.e., FAS assay without malonyl-CoA) were analyzed in parallel. Fragments were identified based on [92].

#### Image acquisition and analysis

Images of GUVs labeled with Fast DiO and live cells were obtained using Yokogawa CSU-X1 spinning disk confocal system mounted on the Eclipse Ti-E Inverted microscope with Nikon CFI Plan Apo Lambda 100X Oil N.A. = 1.45 oil objective, 600 series SS 488nm, SS 561nm lasers and Andor iXon Ultra U3-888-BV monochrome EMCCD camera controlled by Andor IQ3. Maximum intensity projections of 8–10 z stacks of 0.5 μm step size images are shown in Figures 4D, 4E, and S5G. For Figures 5C, 5D, S5D, and S5E single plane images are presented. Fluorescence images are shown with inverted LUT (look-up table).

Image processing and quantifications were performed in Fiji [67]. Images within each experiment images are shown within the same display range. Calibration bars are included. For Figure 4D average fluorescence intensity was measured per cell and normalized to the control. To measure the number of Golgi cisternae (Figure 4E), maximum intensity projections of spinning disk confocal images were thresholded. Cisternae numbers were then normalized to cell area.

#### Measurements of membrane lipid order *in vivo*

Cells were grown in YES medium at 30°C overnight and diluted to OD_595_ 0.2. Following resuspension in fresh medium containing 5 μM di-4-ANEPPDHQ, cells were incubated for 1 hour at 30°C. Cells were then resuspended in fresh medium and transferred into a glass-bottomed microscope dish. Imaging was performed on a Zeiss LSM 780 confocal microscope equipped with a 32 element GaAsP Quasar detector. A 488 nm laser was selected for fluorescence excitation of di-4-ANEPPDHQ. The detection windows were set to 510–580 nm and 620–750 nm. The images were analyzed using a plug-in compatible with Fiji/ImageJ [23].

#### RNA isolation

*S. pombe* and *S. pombe fas* ^*S. j.*^ were grown overnight at 24°C or 30°C to OD_595_ 0.4. 10 OD units of cells were collected and subjected to total RNA extraction using a QIAGEN RNeasy Plus Mini Kit.

#### RNA sequencing

Strand-specific mRNA-seq libraries for the Illumina platform were generated and sequenced at the Advanced Sequencing Facility at the Francis Crick Institute. mRNA libraries were prepared using polyA KAPA mRNA Hyper_Prep with the subsequent quality control using Agilent Technology TapeStation system. A 50-cycle single-read sequence run was performed on the HiSeq 2500 Illumina instrument. Raw sequence data was mapped to the *S. pombe* genome using a Galaxy server pipeline (https://usegalaxy.org/). Quality control was performed using the FastQ Groomer module, reads were mapped to the fission yeast genome using the Tophat module and differential gene expression was assessed by the Cuffdiff module. Functional enrichment analyses were performed using AnGeLi [69]. Genes with fold change log2 values higher than 0.8 or lower than –0.8 and p values < 0.05 were considered for the subsequent analyses. Lists of differentially expressed genes are presented in Data S1, Table 7 (for cells grown at 24°C) and Table 8 (for cells grown at 30°C).

#### Reverse Transcription and Real-Time Quantitative PCR (RT-qPCR)

Reverse transcription was performed at 50°C for 1 hour in a total reaction volume of 20 μL containing 2 μg RNA, 0.5 μg oligo (dT) or hexamer primers with a Transcriptor First Strand cDNA Synthesis Kit (Roche). qPCRBIO Probe Blue Mix Lo-ROX was used for the real-time qPCR assay with the primers generated using Primer3 software [70]. The real-time qPCR was performed on an Applied BioSystem in three biological and two technical replicates. qRT-PCR signal was normalized to GAPDH mRNA expression levels.

#### Measurements of oxygen consumption

Cells were grown in EMM to early stationary phase (OD595 = 1). Typically, 60 mL of cell culture were centrifuged gently, washed once with fresh EMM and resuspended in 80 mL of EMM. The measurements were made using an HI98193 oximeter equipped with Hl764073 probe (Hanna Instruments). Readings were recorded for 15 minutes.

#### Acid phosphatase secretion assay

Cell were grown in EMM until mid-log phase, washed with EMM and diluted to OD_595_ 0.1 in fresh medium for further growth at 30°C. Initial samples were measured at the time of resuspension and used as a reference for further measurements which were taken hourly. Typically, 200 μl of conditioned media was mixed with 200 μl of substrate solution (10mM p-nitrophenyl phosphate, 0.1 M sodium acetate, pH 4) and incubated at 30°C for 30 minutes. Reactions were stopped by addition of 200 μl of 1 M sodium hydroxide and absorbance measured at 405 nm [93].

#### Bioinformatics

Fission yeast protein sequences showing 1:1:1:1 relationship in hierarchical orthologous groups were downloaded from the OMA browser (https://omabrowser.org/oma/home/) [71]. Transmembrane helices (TMHs) were predicted by TMHMM 2.0 (http://www.cbs.dtu.dk/services/TMHMM/) [72] using default parameters. Predictions differing from the median TMH length (23 aa) by ≥ 50% (i.e., ≥ 12 aa) were discarded. TMH amino acid composition was analyzed using ProtParam (https://web.expasy.org/protparam/) [73]. Sequences were aligned using the accurate (L-INS-i) option of MAFFT (https://mafft.cbrc.jp/alignment/software/) [74]. Data were analyzed and plotted in R [75]. Lists of OMA groups corresponding to single-TMH *S. pombe* proteins are presented in Data S1, Table 9.

### Quantification and Statistical Analysis

Lipidomics data were obtained from at least 3-5 replicates (biological and technical) and *p*-values were derived using unpaired parametric heteroscedastic t tests. Experiments shown in Figures 1K–1L were performed in three biological replicates and *p*-values were derived using unpaired parametric t test. For Figure 2, unpaired nonparametric Kolmogorov-Smirnov tests was used to compare bending rigidities and phase separation in GUVs. For qPCR data p values were calculated using unpaired parametric t test. Statistical analyses were performed in Prism 7 (GraphPad Software) and R [75].

### Data and Code Availability

The data that support the findings of this study are available from the corresponding author upon request. The authors declare that all data reported in this study are available within the paper and its supplementary information files. The accession number for raw and processed RNA-seq data reported in this paper is NCBI GEO: GSE141579.

## Supplemental Information

Document S1. Figures S1–S5 and Tables S1–S3

Data S1. Lipidomics, Gene Expression, and Bioinformatics Data, Related to STAR Methods All data presented as Excel tables. (i) Detailed lipidomics dataset for comparison of wild type *S. pombe* and *S. japonicus*—whole lipidome species composition. (ii) Detailed lipidomic dataset—species composition of lipid classes. (iii) Average species composition calculated for the four major lipid classes (PC, PE, PI, and PS). (iv) MS/MS fragmentation data for PC, PE, PI, and PS in *S. japonicus*. (v) Average species composition calculated for the four major lipid classes (PC, PE, PI, and PS) and their species composition upon C18:1 and C10:0 supplementation. (vi) Detailed lipidomics dataset for comparison of *S. pombe* wild type, *S. pombe fas*^*s.j.*^ and *S. pombe fas*^*s.j.*^ cells supplemented with oleic acid—whole lipidome species composition. (vii) List of differential expressed genes in *S. pombe* wild type and *S. pombe fas*^*s.j.*^ cells grown at 24°C. (viii) List of differential expressed genes in *S. pombe* wild type and *S. pombe fas*^*s.j.*^ cells grown at 30°C. (ix) Fission yeast 1:1:1:1 OMA groups corresponding to single-TMH *S. pombe* proteins.

Document S2. Article plus Supplemental Information

## References

[1] Harayama T., Riezman H. (2018). Understanding the diversity of membrane lipid composition. Nat. Rev. Mol. Cell Biol..

[2] Bogdanov M., Dowhan W., Vitrac H. (2014). Lipids and topological rules governing membrane protein assembly. Biochim. Biophys. Acta.

[3] van Meer G., Voelker D.R., Feigenson G.W. (2008). Membrane lipids: where they are and how they behave. Nat. Rev. Mol. Cell Biol..

[4] Sezgin E., Levental I., Mayor S., Eggeling C. (2017). The mystery of membrane organization: composition, regulation and roles of lipid rafts. Nat. Rev. Mol. Cell Biol..

[5] Antonny B., Vanni S., Shindou H., Ferreira T. (2015). From zero to six double bonds: phospholipid unsaturation and organelle function. Trends Cell Biol..

[6] Bogdanov M., Sun J., Kaback H.R., Dowhan W. (1996). A phospholipid acts as a chaperone in assembly of a membrane transport protein. J. Biol. Chem..

[7] Russell J.J., Theriot J.A., Sood P., Marshall W.F., Landweber L.F., Fritz-Laylin L., Polka J.K., Oliferenko S., Gerbich T., Gladfelter A. (2017). Non-model model organisms. BMC Biol..

[8] Gu Y., Oliferenko S. (2019). Cellular geometry scaling ensures robust division site positioning. Nat. Commun..

[9] Yam C., He Y., Zhang D., Chiam K.H., Oliferenko S. (2011). Divergent strategies for controlling the nuclear membrane satisfy geometric constraints during nuclear division. Curr. Biol..

[10] Makarova M., Gu Y., Chen J.S., Beckley J.R., Gould K.L., Oliferenko S. (2016). Temporal regulation of lipin activity diverged to account for differences in mitotic programs. Curr. Biol..

[11] Péter M., Glatz A., Gudmann P., Gombos I., Török Z., Horváth I., Vígh L., Balogh G. (2017). Metabolic crosstalk between membrane and storage lipids facilitates heat stress management in Schizosaccharomyces pombe. PLoS ONE.

[12] Shui G., Guan X.L., Low C.P., Chua G.H., Goh J.S., Yang H., Wenk M.R. (2010). Toward one step analysis of cellular lipidomes using liquid chromatography coupled with mass spectrometry: application to Saccharomyces cerevisiae and Schizosaccharomyces pombe lipidomics. Mol. Biosyst..

[13] Ejsing C.S., Sampaio J.L., Surendranath V., Duchoslav E., Ekroos K., Klemm R.W., Simons K., Shevchenko A. (2009). Global analysis of the yeast lipidome by quantitative shotgun mass spectrometry. Proc. Natl. Acad. Sci. USA.

[14] Stukey J.E., McDonough V.M., Martin C.E. (1990). The OLE1 gene of Saccharomyces cerevisiae encodes the delta 9 fatty acid desaturase and can be functionally replaced by the rat stearoyl-CoA desaturase gene. J. Biol. Chem..

[15] Rhind N., Chen Z., Yassour M., Thompson D.A., Haas B.J., Habib N., Wapinski I., Roy S., Lin M.F., Heiman D.I. (2011). Comparative functional genomics of the fission yeasts. Science.

[16] Moche M., Schneider G., Edwards P., Dehesh K., Lindqvist Y. (1999). Structure of the complex between the antibiotic cerulenin and its target, beta-ketoacyl-acyl carrier protein synthase. J. Biol. Chem..

[17] Elani Y., Purushothaman S., Booth P.J., Seddon J.M., Brooks N.J., Law R.V., Ces O. (2015). Measurements of the effect of membrane asymmetry on the mechanical properties of lipid bilayers. Chem. Commun. (Camb.).

[18] Bigay J., Antonny B. (2012). Curvature, lipid packing, and electrostatics of membrane organelles: defining cellular territories in determining specificity. Dev. Cell.

[19] Baumgart T., Hunt G., Farkas E.R., Webb W.W., Feigenson G.W. (2007). Fluorescence probe partitioning between Lo/Ld phases in lipid membranes. Biochim. Biophys. Acta.

[20] Kastaniotis A.J., Autio K.J., Keratar J.M., Monteuuis G., Makela A.M., Nair R.R., Pietikainen L.P., Shvetsova A., Chen Z., Hiltunen J.K. (2017). Mitochondrial fatty acid synthesis, fatty acids and mitochondrial physiology. Biochim. Biophys. Acta Mol. Cell Biol. Lipids.

[21] Jenni S., Leibundgut M., Boehringer D., Frick C., Mikolásek B., Ban N. (2007). Structure of fungal fatty acid synthase and implications for iterative substrate shuttling. Science.

[22] Wieland F., Renner L., Verfürth C., Lynen F. (1979). Studies on the multi-enzyme complex of yeast fatty-acid synthetase. Reversible dissociation and isolation of two polypeptide chains. Eur. J. Biochem..

[23] Owen D.M., Rentero C., Magenau A., Abu-Siniyeh A., Gaus K. (2011). Quantitative imaging of membrane lipid order in cells and organisms. Nat. Protoc..

[24] Schjerling C.K., Hummel R., Hansen J.K., Borsting C., Mikkelsen J.M., Kristiansen K., Knudsen J. (1996). Disruption of the gene encoding the acyl-CoA-binding protein (ACB1) perturbs acyl-CoA metabolism in Saccharomyces cerevisiae. J. Biol. Chem..

[25] Burr R., Stewart E.V., Shao W., Zhao S., Hannibal-Bach H.K., Ejsing C.S., Espenshade P.J. (2016). Mga2 transcription factor regulates an oxygen-responsive lipid homeostasis pathway in fission yeast. J. Biol. Chem..

[26] Elliott S., Chang C.W., Schweingruber M.E., Schaller J., Rickli E.E., Carbon J. (1986). Isolation and characterization of the structural gene for secreted acid phosphatase from Schizosaccharomyces pombe. J. Biol. Chem..

[27] Kimmig P., Diaz M., Zheng J., Williams C.C., Lang A., Aragón T., Li H., Walter P. (2012). The unfolded protein response in fission yeast modulates stability of select mRNAs to maintain protein homeostasis. eLife.

[28] Guydosh N.R., Kimmig P., Walter P., Green R. (2017). Regulated Ire1-dependent mRNA decay requires no-go mRNA degradation to maintain endoplasmic reticulum homeostasis in *S. pombe*. eLife.

[29] Jackson C.L., Walch L., Verbavatz J.M. (2016). Lipids and their trafficking: an integral part of cellular organization. Dev. Cell.

[30] Vjestica A., Tang X.Z., Oliferenko S. (2008). The actomyosin ring recruits early secretory compartments to the division site in fission yeast. Mol. Biol. Cell.

[31] Sharpe H.J., Stevens T.J., Munro S. (2010). A comprehensive comparison of transmembrane domains reveals organelle-specific properties. Cell.

[32] Lorent J.H., Diaz-Rohrer B., Lin X., Spring K., Gorfe A.A., Levental K.R., Levental I. (2017). Structural determinants and functional consequences of protein affinity for membrane rafts. Nat. Commun..

[33] Quiroga R., Trenchi A., González Montoro A., Valdez Taubas J., Maccioni H.J. (2013). Short transmembrane domains with high-volume exoplasmic halves determine retention of Type II membrane proteins in the Golgi complex. J. Cell Sci..

[34] Jungmann J., Rayner J.C., Munro S. (1999). The Saccharomyces cerevisiae protein Mnn10p/Bed1p is a subunit of a Golgi mannosyltransferase complex. J. Biol. Chem..

[35] He Y., Yam C., Pomraning K., Chin J.S., Yew J.Y., Freitag M., Oliferenko S. (2014). Increase in cellular triacylglycerol content and emergence of large ER-associated lipid droplets in the absence of CDP-DG synthase function. Mol. Biol. Cell.

[36] Manford A.G., Stefan C.J., Yuan H.L., Macgurn J.A., Emr S.D. (2012). ER-to-plasma membrane tethering proteins regulate cell signaling and ER morphology. Dev. Cell.

[37] Roux A., Cuvelier D., Nassoy P., Prost J., Bassereau P., Goud B. (2005). Role of curvature and phase transition in lipid sorting and fission of membrane tubules. EMBO J..

[38] Hui S.W., Mason J.T., Huang C. (1984). Acyl chain interdigitation in saturated mixed-chain phosphatidylcholine bilayer dispersions. Biochemistry.

[39] Xu H., Huang C.H. (1987). Scanning calorimetric study of fully hydrated asymmetric phosphatidylcholines with one acyl chain twice as long as the other. Biochemistry.

[40] Schram V., Thompson T.E. (1995). Interdigitation does not affect translational diffusion of lipids in liquid crystalline bilayers. Biophys. J..

[41] Ali S., Smaby J.M., Momsen M.M., Brockman H.L., Brown R.E. (1998). Acyl chain-length asymmetry alters the interfacial elastic interactions of phosphatidylcholines. Biophys. J..

[42] Aguilar P.S., de Mendoza D. (2006). Control of fatty acid desaturation: a mechanism conserved from bacteria to humans. Mol. Microbiol..

[43] Shimizu I., Katsuki H. (1975). Effect of temperature on ergosterol biosynthesis in yeast. J. Biochem..

[44] Meyer F., Bloch K. (1963). Metabolism of stearolic acid in yeast. J. Biol. Chem..

[45] Proudlock J.W., Haslam J.M., Linnane A.W. (1971). Biogenesis of mitochondria. 19. The effects of unsaturated fatty acid depletion on the lipid composition and energy metabolism of a fatty acid desaturase mutant of Saccharomyces cerevisiae. J. Bioenerg..

[46] Bulder C. (1963). On Respiratory Deficiency in Yeasts. Volume PhD.

[47] Kaino T., Tonoko K., Mochizuki S., Takashima Y., Kawamukai M. (2018). Schizosaccharomyces japonicus has low levels of CoQ_10_ synthesis, respiration deficiency, and efficient ethanol production. Biosci. Biotechnol. Biochem..

[48] Okamoto S., Furuya K., Nozaki S., Aoki K., Niki H. (2013). Synchronous activation of cell division by light or temperature stimuli in the dimorphic yeast Schizosaccharomyces japonicus. Eukaryot. Cell.

[49] Kinnaer C., Dudin O., Martin S.G. (2019). Yeast-to-hypha transition of Schizosaccharomyces japonicus in response to environmental stimuli. Mol. Biol. Cell.

[50] Manni M.M., Tiberti M.L., Pagnotta S., Barelli H., Gautier R., Antonny B. (2018). Acyl chain asymmetry and polyunsaturation of brain phospholipids facilitate membrane vesiculation without leakage. eLife.

[51] Sampaio J.L., Gerl M.J., Klose C., Ejsing C.S., Beug H., Simons K., Shevchenko A. (2011). Membrane lipidome of an epithelial cell line. Proc. Natl. Acad. Sci. USA.

[52] Lande M.B., Donovan J.M., Zeidel M.L. (1995). The relationship between membrane fluidity and permeabilities to water, solutes, ammonia, and protons. J. Gen. Physiol..

[53] Mathai J.C., Tristram-Nagle S., Nagle J.F., Zeidel M.L. (2008). Structural determinants of water permeability through the lipid membrane. J. Gen. Physiol..

[54] Ballweg S., Sezgin E., Wunnicke D., Hänelt I., Ernst R. (2019). Regulation of lipid saturation without sensing membrane fluidity. bioRxiv.

[55] Jiang Y., Vasconcelles M.J., Wretzel S., Light A., Martin C.E., Goldberg M.A. (2001). MGA2 is involved in the low-oxygen response element-dependent hypoxic induction of genes in Saccharomyces cerevisiae. Mol. Cell. Biol..

[56] Heil C.S., Wehrheim S.S., Paithankar K.S., Grininger M. (2019). Fatty acid biosynthesis: chain-length regulation and control. ChemBioChem.

[57] Hofbauer H.F., Schopf F.H., Schleifer H., Knittelfelder O.L., Pieber B., Rechberger G.N., Wolinski H., Gaspar M.L., Kappe C.O., Stadlmann J. (2014). Regulation of gene expression through a transcriptional repressor that senses acyl-chain length in membrane phospholipids. Dev. Cell.

[58] Saitoh S., Takahashi K., Nabeshima K., Yamashita Y., Nakaseko Y., Hirata A., Yanagida M. (1996). Aberrant mitosis in fission yeast mutants defective in fatty acid synthetase and acetyl CoA carboxylase. J. Cell Biol..

[59] Chapman R.E., Munro S. (1994). The functioning of the yeast Golgi apparatus requires an ER protein encoded by ANP1, a member of a new family of genes affecting the secretory pathway. EMBO J..

[60] Balogh G., Péter M., Glatz A., Gombos I., Török Z., Horváth I., Harwood J.L., Vígh L. (2013). Key role of lipids in heat stress management. FEBS Lett..

[61] Török Z., Crul T., Maresca B., Schütz G.J., Viana F., Dindia L., Piotto S., Brameshuber M., Balogh G., Péter M. (2014). Plasma membranes as heat stress sensors: from lipid-controlled molecular switches to therapeutic applications. Biochim. Biophys. Acta.

[62] Cho H., Stanzione F., Oak A., Kim G.H., Yerneni S., Qi L., Sum A.K., Chan C. (2019). Intrinsic structural features of the human IRE1alpha transmembrane domain sense membrane lipid saturation. Cell Rep..

[63] Halbleib K., Pesek K., Covino R., Hofbauer H.F., Wunnicke D., Hanelt I., Hummer G., Ernst R. (2017). Activation of the unfolded protein response by lipid bilayer stress. Mol. Cell.

[64] Mitchell D.C. (2012). Progress in understanding the role of lipids in membrane protein folding. Biochim. Biophys. Acta.

[65] Herzog R., Schwudke D., Schuhmann K., Sampaio J.L., Bornstein S.R., Schroeder M., Shevchenko A. (2011). A novel informatics concept for high-throughput shotgun lipidomics based on the molecular fragmentation query language. Genome Biol..

[66] Behrends V., Tredwell G.D., Bundy J.G. (2011). A software complement to AMDIS for processing GC-MS metabolomic data. Anal. Biochem..

[67] Schindelin J., Arganda-Carreras I., Frise E., Kaynig V., Longair M., Pietzsch T., Preibisch S., Rueden C., Saalfeld S., Schmid B. (2012). Fiji: an open-source platform for biological-image analysis. Nat. Methods.

[68] Geissmann Q. (2013). OpenCFU, a new free and open-source software to count cell colonies and other circular objects. PLoS ONE.

[69] Bitton D.A., Schubert F., Dey S., Okoniewski M., Smith G.C., Khadayate S., Pancaldi V., Wood V., Bähler J. (2015). AnGeLi: a tool for the analysis of gene lists from fission yeast. Front. Genet..

[70] Untergasser A., Cutcutache I., Koressaar T., Ye J., Faircloth B.C., Remm M., Rozen S.G. (2012). Primer3--new capabilities and interfaces. Nucleic Acids Res..

[71] Altenhoff A.M., Glover N.M., Train C.M., Kaleb K., Warwick Vesztrocy A., Dylus D., de Farias T.M., Zile K., Stevenson C., Long J. (2018). The OMA orthology database in 2018: retrieving evolutionary relationships among all domains of life through richer web and programmatic interfaces. Nucleic Acids Res..

[72] Krogh A., Larsson B., von Heijne G., Sonnhammer E.L. (2001). Predicting transmembrane protein topology with a hidden Markov model: application to complete genomes. J. Mol. Biol..

[73] Gasteiger E., Hoogland C., Gattiker A., Duvaud S., Wilkins M.R., Appel R.D., Bairoch A. (2005). The Proteomics Protocols Handbook.

[74] Katoh K., Standley D.M. (2013). MAFFT multiple sequence alignment software version 7: improvements in performance and usability. Mol. Biol. Evol..

[75] Team R.D.C. (2008). R: A Language and Environment for Statistical Computing.

[76] Moreno S., Klar A., Nurse P. (1991). Molecular genetic analysis of fission yeast Schizosaccharomyces pombe. Methods Enzymol..

[77] Furuya K., Niki H. (2009). Isolation of heterothallic haploid and auxotrophic mutants of Schizosaccharomyces japonicus. Yeast.

[78] Aoki K., Nakajima R., Furuya K., Niki H. (2010). Novel episomal vectors and a highly efficient transformation procedure for the fission yeast Schizosaccharomyces japonicus. Yeast.

[79] Bähler J., Wu J.Q., Longtine M.S., Shah N.G., McKenzie A., Steever A.B., Wach A., Philippsen P., Pringle J.R. (1998). Heterologous modules for efficient and versatile PCR-based gene targeting in Schizosaccharomyces pombe. Yeast.

[80] Alsabeeh N., Chausse B., Kakimoto P.A., Kowaltowski A.J., Shirihai O. (2018). Cell culture models of fatty acid overload: Problems and solutions. Biochimica et biophysica acta. Mol. Cell Biol. Lipids.

[81] Liebisch G., Binder M., Schifferer R., Langmann T., Schulz B., Schmitz G. (2006). High throughput quantification of cholesterol and cholesteryl ester by electrospray ionization tandem mass spectrometry (ESI-MS/MS). Biochim. Biophys. Acta.

[82] Murphy R.C. (2014). Tandem Mass Spectrometry of Lipids: Molecular Analysis of Complex Lipids.

[83] Liebisch G., Vizcaíno J.A., Köfeler H., Trötzmüller M., Griffiths W.J., Schmitz G., Spener F., Wakelam M.J. (2013). Shorthand notation for lipid structures derived from mass spectrometry. J. Lipid Res..

[84] Balogh G., Péter M., Liebisch G., Horváth I., Török Z., Nagy E., Maslyanko A., Benko S., Schmitz G., Harwood J.L., Vígh L. (2010). Lipidomics reveals membrane lipid remodelling and release of potential lipid mediators during early stress responses in a murine melanoma cell line. Biochim. Biophys. Acta.

[85] López Mora N., Hansen J.S., Gao Y., Ronald A.A., Kieltyka R., Malmstadt N., Kros A. (2014). Preparation of size tunable giant vesicles from cross-linked dextran(ethylene glycol) hydrogels. Chem. Commun. (Camb.).

[86] Karamdad K., Law R.V., Seddon J.M., Brooks N.J., Ces O. (2016). Studying the effects of asymmetry on the bending rigidity of lipid membranes formed by microfluidics. Chem. Commun. (Camb.).

[87] Pécréaux J., Döbereiner H.G., Prost J., Joanny J.F., Bassereau P. (2004). Refined contour analysis of giant unilamellar vesicles. Eur Phys J E Soft Matter.

[88] Yoon Y.Z., Hale J.P., Petrov P.G., Cicuta P. (2010). Mechanical properties of ternary lipid membranes near a liquid-liquid phase separation boundary. J. Phys. Condensed Matter.

[89] Bhatia T., Husen P., Brewer J., Bagatolli L.A., Hansen P.L., Ipsen J.H., Mouritsen O.G. (2015). Preparing giant unilamellar vesicles (GUVs) of complex lipid mixtures on demand: Mixing small unilamellar vesicles of compositionally heterogeneous mixtures. Biochim. Biophys. Acta.

[90] Fischer M., Rhinow D., Zhu Z., Mills D.J., Zhao Z.K., Vonck J., Grininger M. (2015). Cryo-EM structure of fatty acid synthase (FAS) from Rhodosporidium toruloides provides insights into the evolutionary development of fungal FAS. Protein Sci..

[91] Lynen F. (1969). Yeast Fatty Acid Synthase.

[92] Haynes C.A., Allegood J.C., Sims K., Wang E.W., Sullards M.C., Merrill A.H. (2008). Quantitation of fatty acyl-coenzyme As in mammalian cells by liquid chromatography-electrospray ionization tandem mass spectrometry. J. Lipid Res..

[93] Wang H., Tang X., Liu J., Trautmann S., Balasundaram D., McCollum D., Balasubramanian M.K. (2002). The multiprotein exocyst complex is essential for cell separation in Schizosaccharomyces pombe. Mol. Biol. Cell.

