# Supplementary material for: Delineating the Rules for Structural Adaptation of Membrane-Associated Proteins to Evolutionary Changes in Membrane Lipidome
Source: Curr Biol. 2020 Feb 3;30(3):367–380.e8. doi: 10.1016/j.cub.2019.11.043 (PMC6997885; doi:10.1016/j.cub.2019.11.043)
Supplement: Document S1. Figures S1–S5 and Tables S1–S3 [file mmc1.pdf]

Current Biology, Volume *30*

## Supplemental Information

# Delineating the Rules for Structural Adaptation of Membrane-Associated Proteins to Evolutionary Changes in Membrane Lipidome

**Maria Makarova, Maria Peter, Gabor Balogh, Attila Glatz, James I. MacRae, Nestor Lopez Mora, Paula Booth, Eugene Makeyev, Laszlo Vigh, and Snezhana Oliferenko**

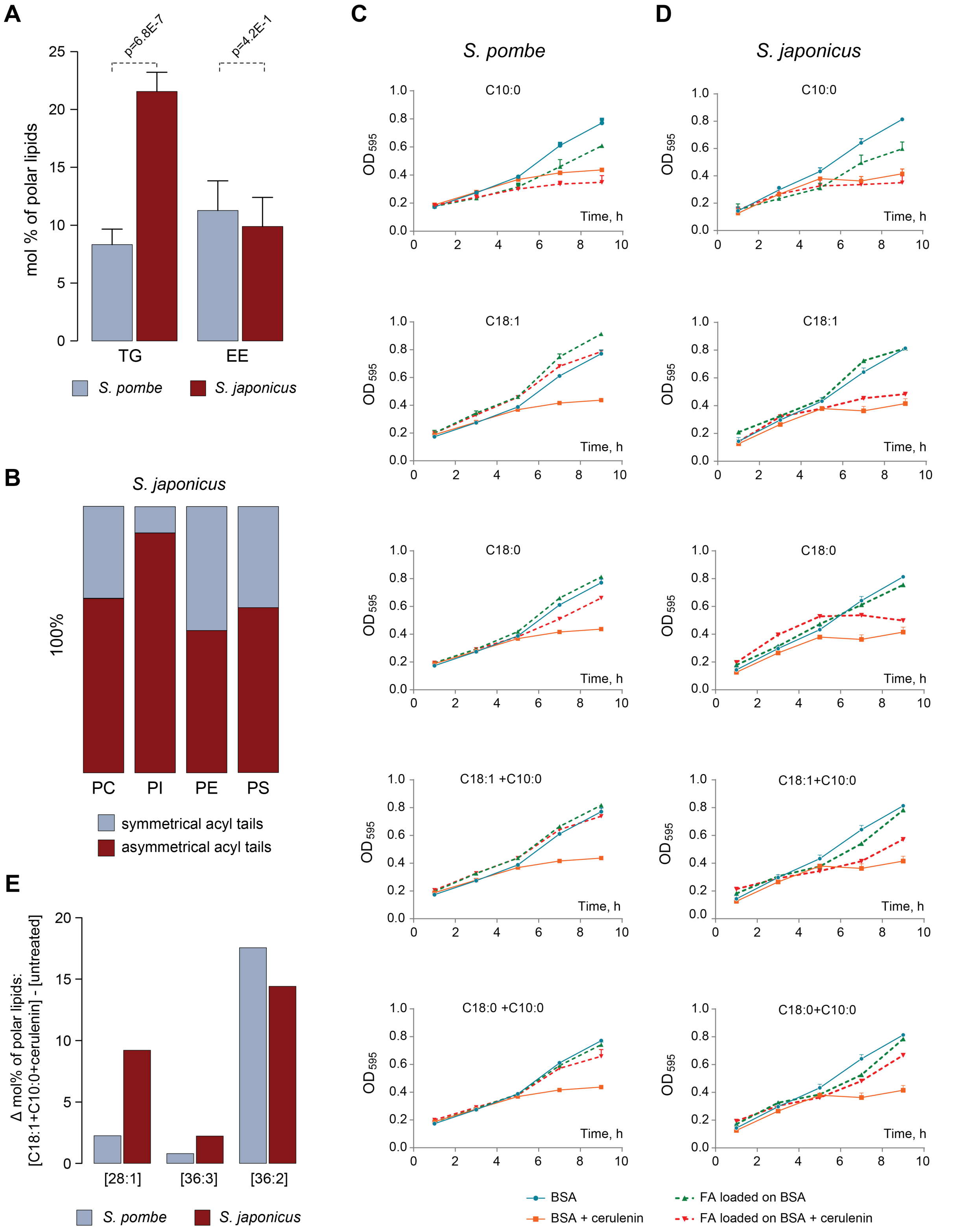


**Figure S1. *S. japonicus* efficiently incorporates C10:0 FA into glycerophospholipids and requires it for growth, related to Figure 1.** (**A**) Relative abundance of triacylglycerol (TG) and sterol esters (EE) in *S. pombe* and *S. japonicus*. Shown are the mean values ± SD ($n$ = 5). $p$ values derived from the unpaired parametric t-test. (**B**) A graph representing percentages of 'asymmetrical'

molecular species (defined as GPLs with acyl chains that differ in length more than 6 carbons) for the four indicated lipid classes in *S. japonicus*, calculated from the mass-spectrometry fragmentation analysis. Growth curves for *S. pombe* (**C**) and *S. japonicus* (**D**) cultures used to derive growth rates presented in Figure 1K. (**C**-**D**) Shown are the mean values ± SD per time point ($n$ = 3). (**E**) A graph representing the average differences Δ (in mol% of PC, PI, PE, PS) of the indicated GPL species, between the wild type *S. pombe* and *S. japonicus* and cells grown in the presence of cerulenin, C18:1 and C10:0 FAs. Cells were treated for 8 hours. Species' enrichment in the membranes of treated cells is indicated. Supplementation with C18:1 and C10:0 primarily resulted in the enrichment of two GPL species, 28:1 (18:1/10:0) and 36:2 (di-18:1), as well as 36:3 (18:1/18:2) which can be formed from C18:1 via an additional desaturation step. Note that the ratio of 28:1/36:2+36:3 was 0.55 in *S. japonicus* and 0.12 in *S. pombe*, suggesting that C10:0 was incorporated in *S. japonicus* membrane lipids ~ 4.6 times more efficiently than in *S. pombe*. $n$ = 3.

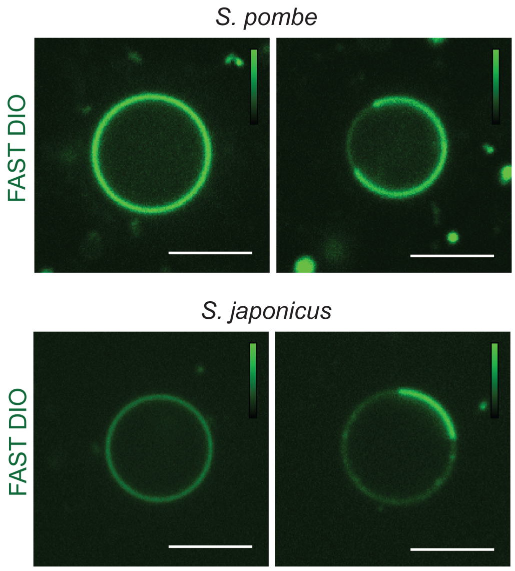


**Figure S2. Examples of the primary microscopy data used to calculate Ld/Lo values, related to Figure 2.** Note differences in FAST DIO staining intensities between GUVs formed from *S. pombe* and *S. japonicus* total polar lipids – this dye preferentially partitions to Ld membranes. The summary of this experiment is shown in Figure 2B. Intensity calibration bars are included. Scale bar, 5 μm.

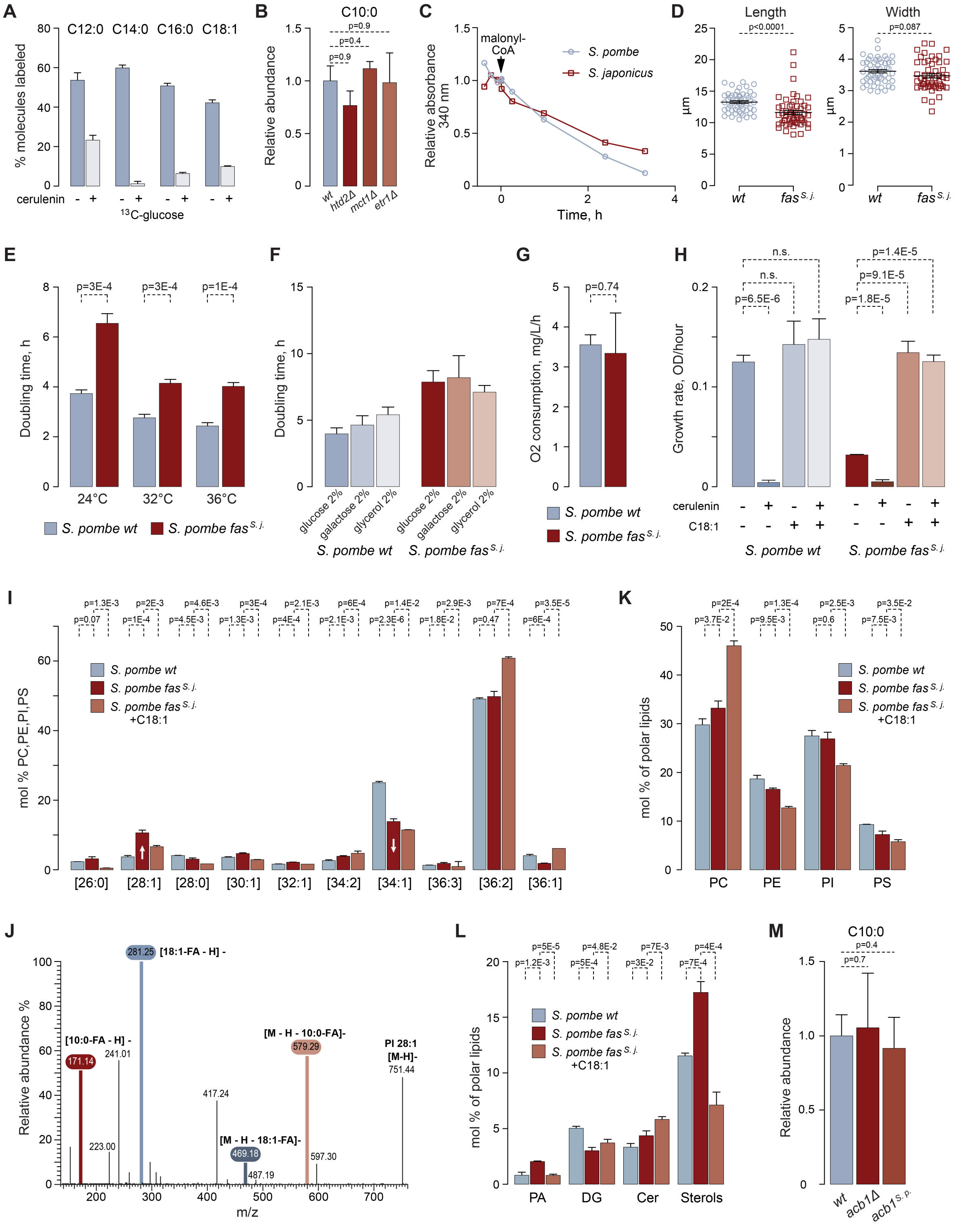


**Figure S3. *S. japonicus* cytosolic FAS complex produces C10:0 FA, related to Figure 3.** (**A**) A plot describing the results of the GC-MS analysis of indicated $^{13}C$-labeled FAs extracted from *S. japonicus* cells grown in the presence of the U-$^{13}C$-

glucose for 4 hours. 10 μM cerulenin was added when indicated. Shown are the means of percentages of $^{13}$C-labeled FAs ± SD (n = 6). (**B**) Relative abundance of C10:0 in the wild type and three mutants in the mitochondrial FA synthesis pathway. Genotypes of mutants are indicated. (**C**) A line chart tracing relative absorbance of NADPH at 340 nm in protein fractions used for *in vitro* FA synthesis reactions shown in Figure 3C. Malonyl-CoA was added at time 0. (**D**) Quantifications of cell length and width in cells of indicated genotypes (n=52). Means are shown. *p* values derived from unpaired t-test. (**E**) Doubling times for *S. pombe* wild type and $fas^{s.\ j.}$ cultures grown at indicated temperatures in the EMM medium. (**F**) Doubling times for *S. pombe* wild type and $fas^{s.\ j.}$ cultures grown at 32°C in the YE-based media with indicated carbon sources (*n = 3*). (**G**) Oxygen consumption of *S. pombe* wild type and $fas^{s.\ j.}$ cultures grown in EMM at 32°C. (**H**) Growth rates of *S. pombe* wild type and $fas^{s.\ j.}$ cultures in the presence or the absence of cerulenin and C18:1 FA. (**E**, **G**, **H**) Shown are the mean values ± SD (n = 3). *p* values derived from the unpaired parametric t-test. (**I**) A graph representing the average molecular species profiles for the four major GPLs in *S. pombe* wild type, $fas^{s.\ j.}$ and $fas^{s.\ j.}$ cells grown in the presence of C18:1. Phospholipids are specified by the total carbon atoms: total double bonds in acyls. Note an increase in the 28:1 at the expense of 34:1 species in *S. pombe* $fas^{s.\ j.}$ cells. The 28:1 species decreased when cells were supplemented with C18:1. Shown are the mean values ± SD (*n* = 3). *p* values derived from the unpaired parametric t-test. (**J**) A representative mass spectrum of the PI 28:1 molecular species from the *S. pombe* $fas^{s.\ j.}$ lipid extract indicating ions generated upon fragmentation. Graphs representing the abundance of the four main GPL classes (PC, PI, PE, and PS) (**K**) and other indicated membrane lipids (**L**) in *S. pombe* wild type, $fas^{s.\ j.}$ and $fas^{s.\ j.}$ cells grown in the presence of C18:1, presented as molecular percentages of membrane lipids. (**M**) Relative abundance of C10:0 in the wild type and *acb1* mutants of indicated genotypes. (**B**, **M**) Shown are the means of relative C10:0 content ± SD (n = 6). *p* values derived from the unpaired parametric t-test. (**I, K-L**) Shown are the mean values ± SD (*n* = 3). *p* values derived from the unpaired parametric t-test.

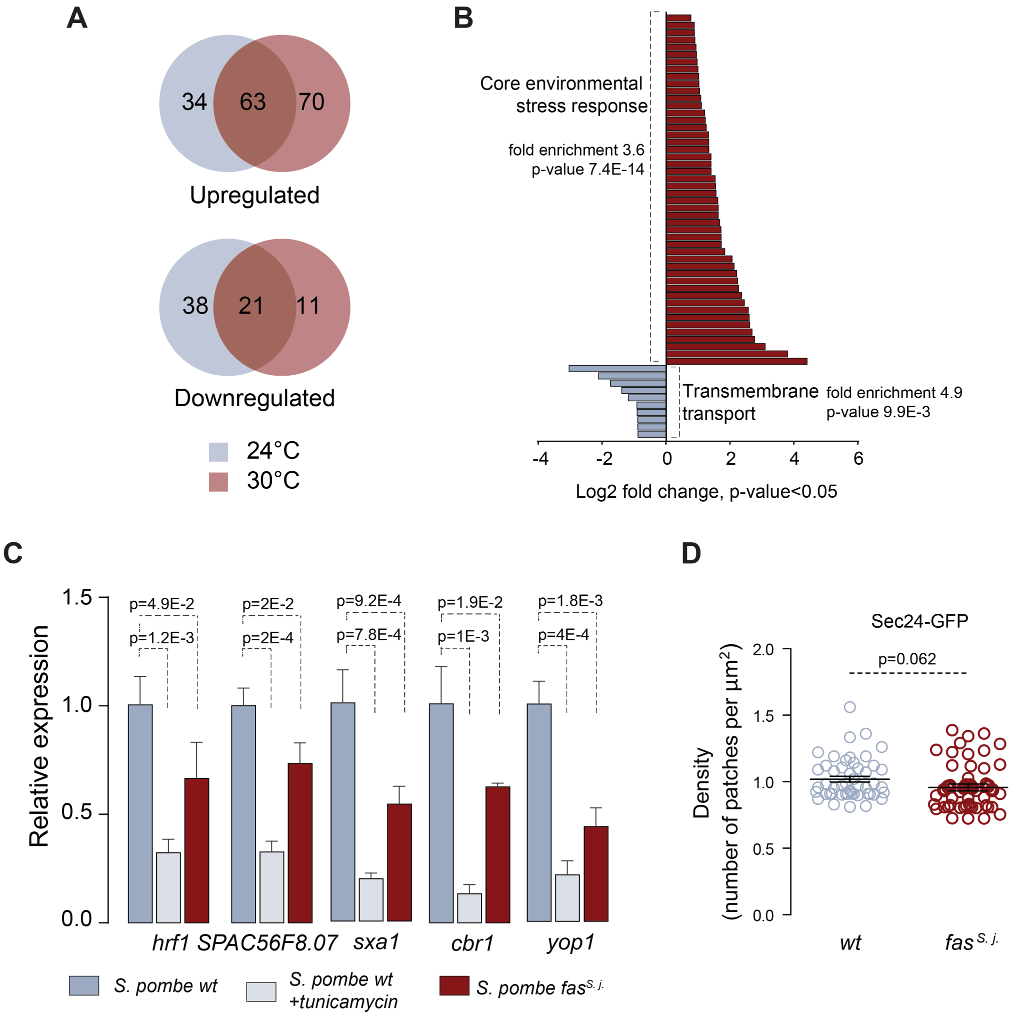


**Figure S4. Replacement of *S. pombe* FAS with the *S. japonicus* version causes environmental stress response and activates UPR, related to Figure 4.** (**A**) Venn diagrams showing an overlap between the differentially expressed genes in wild type and *fas*$^{s.\ j.}$ S. *pombe* cells, at 24°C and 30°C. (**B**) Differentially expressed genes constituting two largest functional categories in *S. pombe fas*$^{s.\ j.}$ cells as compared to the wild type. Cells were grown in YES at 30°C. AnGeLi suite was used for gene category annotations. (**C**) A graph summarizing the results of qPCR analyses of indicated poly-A mRNAs in *S. pombe* wild type, *S. pombe* wild type treated with 0.5 µg/ml tunicamycin for one hour to induce the UPR and *S. pombe fas*$^{s.\ j.}$ mutant cells. Shown are the mean values derived from three biological and two technical repeats, normalized to the wild type. *p* values derived from the unpaired parametric t-test. (**D**) The densities of Sec24-GFP-positive tER structures (numbers per unit of area) in *S. pombe* wild type and *fas*$^{s.\ j.}$ mutant cells, normalized to the wild type. The analysis is related to Figure 4D. Means are shown. *p* values derived from the unpaired parametric t-test.

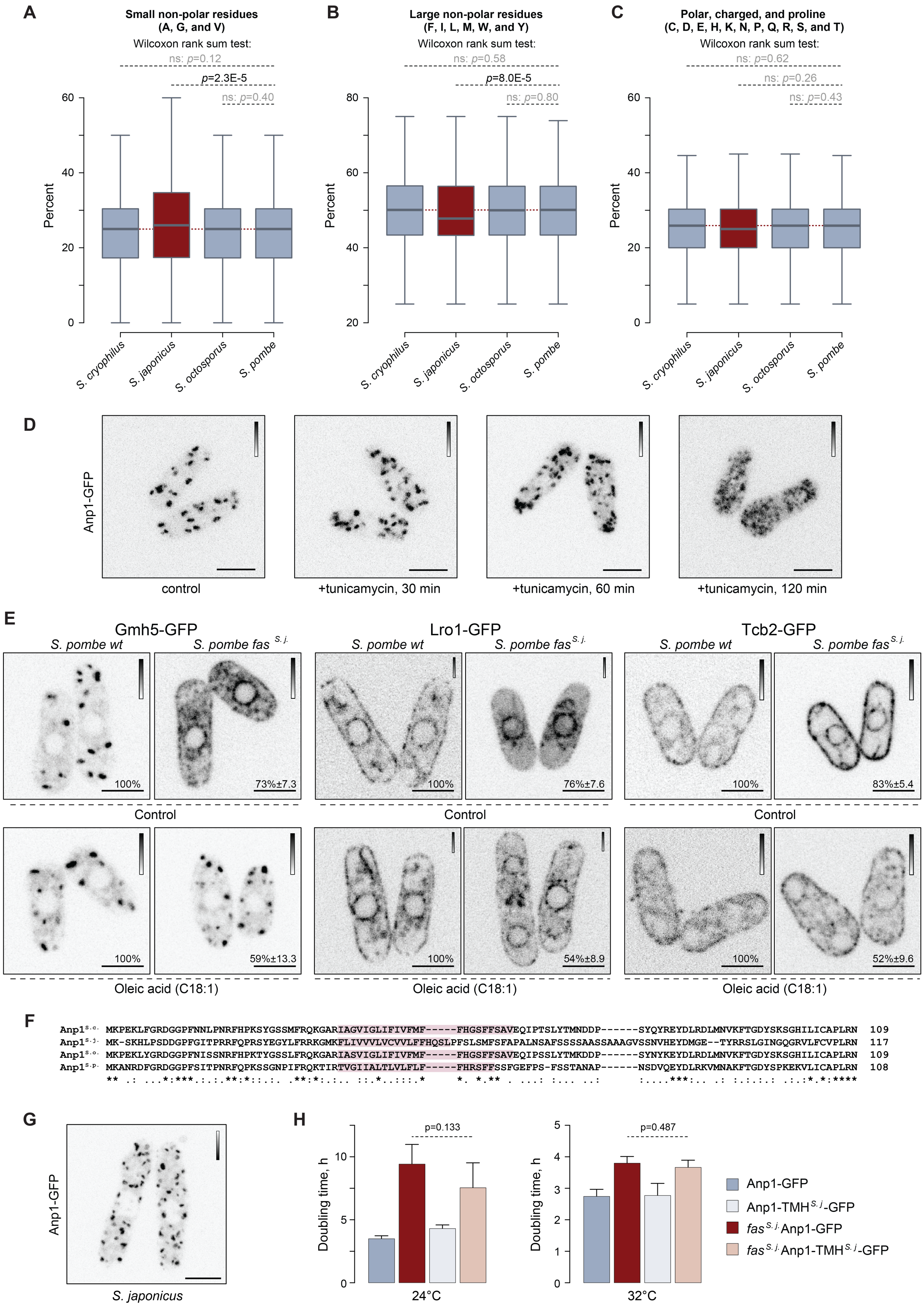


**Figure S5. Transmembrane helices in a number of *S. japonicus* proteins may have adapted to evolutionary changes in membrane lipid composition, related**

**to Figure 5.** Box plots showing distributions of percentages of small non-polar residues (**A**), large non-polar residues (**B**) and the rest of amino acids (**C**) in all predicted TMHs in proteomes of the four fission yeasts. The dashed lines mark positions of the medians in *S. pombe*. Outliers are not shown. *p* values derived from the Wilcoxon rank sum test. (**D**) Single plane spinning-disk confocal images of *S. pombe* cells expressing Anp1-GFP, treated with 0.5 µg/ml tunicamycin for indicated times to induce the UPR. (**E**) Single plane spinning-disk confocal images of cells expressing Gmh5-GFP, Lro1-GFP and Tcb2-GFP in *S. pombe* wild type (n=102, n=78 and n=102 cells, respectively) and *fas*$^{s.\ j.}$ cells (n=73, n=94 and n=103 cells, respectively) in the absence or the presence of C18:1 supplementation (n=105, n=78 and n=104 cells for wild type and n=89, n=86 and n=102 cells for *fas*$^{s.\ j.}$ cells). Included are the percentages of cells in a population exhibiting the indicated phenotypes.(**F**) Multiple sequence alignments for N-terminal portions of Anp1 orthologs from the four fission yeast species including Anp1$^{S.c.}$ (OMA ID SCHCR05123), Anp1$^{S.j.}$ (SCHJY01063), Anp1$^{S.o.}$ (SCHOY00891) and Anp1$^{S.p.}$ (SCHPO02701). "*" corresponds to invariant, ":" to strongly conserved, and "." to weakly conserved residues, according to the Clustal convention. TMHMM-predicted transmembrane helices are highlighted in pink. (**G**) Multiple projection of the spinning-disk confocal z-stack of *S. japonicus* cells expressing Anp1-GFP. (**H**) Doubling times of *S. pombe* cultures of indicated genotypes grown at 24°C and 32°C in the YES medium. Shown are the mean values ± SD (*n = 3*). *p* values derived from the unpaired parametric t-test. (**D**, **E, G**) Scale bar, 5 µm. Calibration bars are shown for each image.

| | |
|---|---|
| 753.46 | parent ion [M-H]- |
| 599.32 | loss of neutral 10:0 ketene; 753-172+18 = 599 |
| 581.31 | loss of neutral 10:0 FA; 753-172 = 581 |
| 553.28 | loss of neutral 12:0 FA; 753-200 = 553 |
| 487.19 | loss of neutral 18:0 ketene; 753-284+18 = 487 |
| 469.18 | loss of neutral 18:0 FA ; 753-284 = 469 |
| 419.26 | loss of neutral 10:0 ketene followed by loss of a neutral inositol epoxide (180 Da); 753-172+18-180 = 419 |
| 391.23 | loss of neutral 12:0 ketene followed by loss of a neutral inositol epoxide (180 Da); 753-200+18-180 = 391 |
| 283.26 | 18:0 carboxylate anion |
| 255.23 | 16:0 carboxylate anion |
| 241.01 | cyclic anion of inositol phosphate |
| 223.00 | water loss from cyclic anion of inositol phosphate |
| 199.17 | 12:0 carboxylate anion |
| 171.14 | 10:0 carboxylate anion |
| 153.00 | water loss from glycerol phosphate anion |

**Table S1. Full assignment of MS/MS fragmentation for PI(28:0). Related to Figure 1.**

| | Parent ion | CoA-specific fragments | | | FA-specific fragments | | |
|---|---|---|---|---|---|---|---|
| FA-CoA | [M-H]- | [pA]- | [pAP-H2O]- | [pAP]- | [M-pAP-H2O]- | [M-pA-H2O]- | [M-pA]- |
| 18:0 | 1032.37 | 328.04 | 408.01 | 426.02 | 605.34 | 685.3 | 703.31 |
| 16:0 | 1004.34 | 328.04 | 408.01 | 426.02 | 577.31 | 657.27 | 675.28 |
| 10:0 | 920.24 | 328.04 | 408.01 | 426.02 | 493.21 | 573.17 | 591.18 |

pA, phospho-adenosine; pAP, phospho-adenosine phosphate

**Table S2. MS/MS fragmentation data for detected FA-CoAs. Related to Figure 3.**

| Sequence, 5' to 3' | Description | Supplier |
|---|---|---|
| TAACTCTTCACTAAATCACGAAACTCGTTAGTTACTACTATCAGTCATTTTAACCTGGCTGGTTGAGCGTGATAGCCGCAGTTTAGCTTGCCTCGTCCCC | htd2_sj kanMX6 cassette knockout fwd | TF |
| CCAACCCGCAAAACTGGACAAGGTTCGCATTCGATGTACCTGCATACTCACGGTTCTTCATTGCTCTTTCGTCATGCACAGAATTCGAGCTCGTTTAAAC | htd2_sj kanMX6 cassette knockout rev | TF |
| AAATAAAAGTCTTTCACCGTATTCGTTTTTTGCCTGTCTTGTTTTCTCTACGGTCGCTTCGTCGCCTAGATCGCTTCATCGTTTAGCTTGCCTCGTCCCC | mct1_sj kanMX6 cassette knockout fwd | TF |
| TTGTGCGTATGTACAGTAGTAGTAATAGTAGTCATAGTGGGCGAGGTTAGGATGTCTCTACGTTGACGTGTATAGTGCAAGAATTCGAGCTCGTTTAAAC | mct1_sj kanMX6 cassette knockout rev | TF |
| AAGGGATAATTCTTTTGTTTTCACTAAAGAAGTTTCTCGTTTGTTTGTTTTCCCACTACACCAGCAACACATCGTCTAATGTTTAGCTTGCCTCGTCCCC | etr1_sj kanMX6 cassette knockout fwd | TF |
| AGTATAGCTACAACGTTAAAGAACACGTTATATATGTTTCATGCATCAACTCGTAAATTCTGGTACTACGAACGTAATTAGAATTCGAGCTCGTTTAAAC | etr11_sj kanMX6 cassette knockout rev | TF |
| TACTGAGACGCCGCTACAAA | htd2_sj ORF genotyping fwd | IDT |
| CTGCTGAGGATCCTGTACGT | htd2_sj ORF genotyping rev | IDT |
| GGTGCATCTCATTAAAGCACTG | htd2_sj 5'UTR genotyping fwd | IDT |
| TCAATACTTCTTTCTAGATGG | htd2_sj 3'UTR genotyping rev | IDT |
| TCTTCGTGTCGTTGATGTGC | mct1_sj ORF genotyping fwd | IDT |
| TTGCAGGGCCATTGTCAAAA | mct1_sj ORF genotyping rev | IDT |
| CGCGGCGGCTACTTATTATT | mct1_sj 5'UTR genotyping fwd | IDT |
| GGTATCATTGAGCCCTCTTT | mct1_sj 3'UTR genotyping rev | IDT |
| CCGGTCAATGGGCTGTTATG | etr1_sj ORF genotyping fwd | IDT |
| ATCAACGTCAGGAGAGTGCA | etr1_sj ORF genotyping rev | IDT |
| TCTGGTAATACTGAACGCTC | etr1_sj 5'UTR genotyping fwd | IDT |
| ATGGAGCGAAAGAGGTGAGT | etr1_sj 3'UTR genotyping rev | IDT |
| TCCCCCGGGTATGGTCACAAACATGGTT | SmaI_acb1_sj 5'UTR to knockout ORF fwd | IDT |
| AGAGGATCCATATCCTTGAGTAAGTCCGA | BamHI_acb1_sj 5'UTR to knockout ORF rev | IDT |
| TCCGGGCCCATAGACACAGTCATTCAGCTC | ApaI_acb1_sj 3'UTR to knockout ORF fwd | IDT |
| TCCCCCGGGGCAAACTGTGCGTTATCG | SmaI_acb1_sj 3'UTR to knockout ORF rev | IDT |
| GTGTAACTACTCTCACGCATTC | acb1_sj ORF genotyping fwd | IDT |
| ACCGTACTTGGACTTGAGCTC | acb1_sj ORF genotyping rev | IDT |
| TTGCAGACACGGAGGCCTGAA | acb1_sj 5'UTR genotyping fwd | IDT |
| ATCTCGTTGACTACATGCTC | acb1_sj 3'UTR genotyping rev | IDT |
| AGAGGATCCATGTCTTCTACTTTCGAGCAAGC | BamHI_acb1_sp ORF cloning fwd | IDT |
| ATAGGATCCTTACTTCATTCCATACTTGGTCT | BamHI_acb1_sp ORF cloning rev | IDT |
| TCTCCCGGGAACTAGCTGTCTAACAGATCACACT | SmaI_fas1_sp 5'UTR to replace ORF fwd | IDT |
| TAGAGGATCCGATTTCAATGATAATATGAGAA | BamHI_fas1_sp 5'UTR to replace ORF rev | IDT |
| AGGAGGGCCCATATAAAGTATAAAGTTATTGG | ApaI_fas1_sp 3'UTR to replace ORF fwd | IDT |
| CAGACCCGGGAAGATGCGTCTTCAAAAGGAAG | SmaI_fas1_sp 3'UTR to replace ORF rev | IDT |
| TAGAGGATCCATGGTGGAGACAGGATATACGAGTCC | BamHI_fas1_sj ORF fwd | IDT |
| TAGATGTGCGGCCGCTTAGGAAGAAGCATAAGTATCCCAG | NotI_fas1_sj ORF rev | IDT |
| CATCCGGTACCTTTACGTTATATTGTCTTTCAAC | KpnI_fas2_sp 5'UTR to replace ORF fwd | IDT |
| GAAGAGGGCCCGTTGCTGTTAGACTCGATGGT | ApaI_fas2_sp 5'UTR to replace ORF rev | IDT |
| GAAGAGGGCCCATCTCACATAGTCGAAATACG | ApaI_fas2_sp 3'UTR to replace ORF fwd | IDT |
| TAGGACCCGGGTATTCAAATGGTTTAACAAAAC | SmaI_fas2_sp 3'UTR to replace ORF rev | IDT |
| TAGGACCCGGGATGAGACCTGAAGTCGAGCAGG | SmaI_fas2_sj ORF fwd | IDT |
| GGCATGCGGCCGCTTAGTGCTCAGCGATAGCAACAG | NotI_fas2_sj ORF rev | IDT |
| CGAGTGAGTCTGACAAGACA | fas1_sj ORF genotyping rev | IDT |
| CACTTGCACAGTTTGAGCAC | fas1_sj ORF genotyping fwd | IDT |
| ATGTGTCGCCATCACAGCCA | fas1_sp 3'UTR genotyping rev | IDT |
| GTGGATCCTACCAATTCCCA | fas1_sp 5'UTR genotyping fwd | IDT |
| GGTAACTTGTTGAGTTTGTC | fas1_sp ORF genotyping fwd | IDT |
| ACCAAATAGTCACGTTCCGG | fas1_sp ORF genotyping rev | IDT |

| | | |
|---|---|---|
| TGCAACTGGCTCAGGAGCTA | fas2_sj ORF genotyping rev | IDT |
| CTGGTTGAAGCTGGGGCTTC | fas2_sp ORF genotyping rev | IDT |
| GATGCTCTTGTCAACAACAA | fas2_sj ORF genotyping fwd | IDT |
| CCACGATGCGCTGGTTTACA | fas2_sp ORF genotyping fwd | IDT |
| CAGCTGTTCCGACAGATTCT | fas2_sp 3'UTR genotyping rev | IDT |
| CGGTGGCAGGTAGATATAAG | fas2_sp 5'UTR genotyping fwd | IDT |
| CTTGCCAAGGTCATCAACGA | SPBC32F12.11 (GAPDH sp) qPCR fwd | IDT |
| AGGGACACGGAAAGCCATAC | SPBC32F12.11 (GAPDH sp) qPCR rev | IDT |
| TACGATTTGCTCCAATGCCG | gas2_sp qPCR fwd | IDT |
| CAGCTACAAGAATCGGCACC | gas2_sp qPCR rev | IDT |
| CAGACACTTGATCCGTCCCT | yop1_sp qPCR fwd | IDT |
| TGCAAAAGAAGAGGCTGTGG | yop1_sp qPCR rev | IDT |
| TAGTACCCCGTTGCATGGAG | SPCC970.03 qPCR fwd | IDT |
| CGTCCACGCCAGAACTTATG | SPCC970.03 qPCR rev | IDT |
| TGTCGATCGCTGCTACTTCT | sxa1_sp qPCR fwd | IDT |
| ATCGAAACGCCAACACTAGC | sxa1_sp qPCR rev | IDT |
| AGCTGTCTTTCTGGTTTGCG | Spac56F8.07 qPCR fwd | IDT |
| AGCATGCACCCCGTATATGA | Spac56F8.07 qPCR rev | IDT |
| CCCGTACTTACAACCGCAAG | bip1_sp qPCR fwd | IDT |
| TTGGGAGGACAAAGTACGCT | bip1_sp qPCR rev | IDT |
| TGGTATCACTTGCTCCGGAA | ire1_sp qPCR fwd | IDT |
| TGAGCCTTCAGATTCCACGT | ire1_sp qPCR rev | IDT |
| CTGCTTTCGATGCTGTTGGA | pho1_sp qPCR fwd | IDT |
| AATAACTCGACACGGCCACT | pho1_sp qPCR rev | IDT |
| TGGCGGTAGAACAGGGATTT | SPBC887.17 qPCR fwd | IDT |
| ATGTAGAACCGGTAGCCCAG | SPBC887.17 qPCR rev | IDT |
| CCCTCCTGCGGAAGACTTAA | hrf1_sp qPCR fwd | IDT |
| AAACGCGTCGCTAGGAATTC | hrf1_sp qPCR rev | IDT |
| GGGACTGCCTAGATGAAATAAATCATTATAAGGGATTAGCGCAA AGAAAACGGGGTTGGAGGAAGTTAGTTCCGTTTCTTCGGATCCC CGGGTTAATTAA | gmh5_sp kanMX6 cassette Cterminal GFP tagging fwd | TF |
| GAGTTTAGTGCATCACATGATAAAGGATCGCATTGACAGCCTAG CACACGCAAGGAAAAAGAATGATAAAGTATGAATTTGAATTCGA GCTCGTTTAAAC | gmh5_sp kanMX6 cassette Cterminal GFP tagging rev | TF |
| GAAGATGGGATGGAGAAATCTTCGAAGAAAATTTGCTTTGAACG GACGACAGCCAGATGTTGAAACCGAGATTTCAAAACGGATCCCC GGGTTAATTAA | tcb2_sp kanMX6 cassette Cterminal GFP tagging fwd | TF |
| ACTTTTATCATGAACAGACCATAAATAATCAAACACACGTATTTA AATCTCGAACACACGGTCAAGAATGTTCTTCCATAGAATTCGAG CTCGTTTAAAC | tcb2_sp kanMX6 cassette Cterminal GFP tagging rev | TF |
| GGAGGGCCCGTCGTTGTAGACCCGAGAAT | ApaI_anp1_sp 5'UTR to replace ORF fwd | IDT |
| GAAGACCCGGGTGTGGAAGAGATTAACAATAc | SmaI_anp1_sp 5'UTR to replace ORF rev | IDT |
| CATCCGGTACCTCCTGAGCACAGTAACCATG | KpnI_anp1_sp 3'UTR to replace ORF fwd | IDT |
| GGAGGGCCCGCTACGTGAGCAGATTACCA | ApaI_anp1_sp 3'UTR to replace ORF rev | IDT |
| AACTAAACCAACCCCGTACC | anp1_sp 5'UTR genotyping fwd | IDT |
| CGCGACCATCATTATATATG | anp1_sp 3’UTR genotyping rev | IDT |
| GCACCGAAATCGATGGAGAA | fasI_sj sequencing rev | IDT |
| GGAGACAGGATATACGAGTC | fasI_sj sequencing fwd | IDT |
| TCTCTCTCCCCCTTATTGGT | fasI_sj sequencing fwd | IDT |
| AGCTCCCTATGATGCTTGCT | fasI_sj sequencing fwd | IDT |
| CAACTCCGTCATCTCCATTG | fasI_sj sequencing fwd | IDT |
| GGACAAGCGTTGGATTGACA | fasI_sj sequencing fwd | IDT |
| ATCGCCAAGAACTGGAAGCT | fasI_sj sequencing fwd | IDT |
| AACGGCACTGATGTCTTGGA | fasI_sj sequencing fwd | IDT |
| CCCAACTCTGAGCTTGTTAC | fasI_sj sequencing fwd | IDT |
| AATACGGTATGGTTGCCGCA | fasI_sj sequencing fwd | IDT |
| ATCCATCTGACGGGAGATG | fasII_sj sequencing rev | IDT |
| TGAAGTCGAGCAGGAATTGG | fasII_sj sequencing fwd | IDT |
| AAGCTAAGGCTTGGCTTGAC | fasII_sj sequencing fwd | IDT |
| ATGAAGAGGTTCCTCGCTCT | fasII_sj sequencing fwd | IDT |
| GAGTTCTGAGTCTTGGTCGA | fasII_sj sequencing fwd | IDT |

| | | |
|---|---|---|
| CCCGTCGATGATAAGGATGT | fasII_sj sequencing fwd | IDT |
| GAATGAGTGTGATGTCCTCG | fasII_sj sequencing fwd | IDT |
| AATGATCTCCTCCGGTAAGG | fasII_sj sequencing fwd | IDT |

**Table S3. List of primers used in this study. Related to STAR Methods.** TF, Thermo Fisher Scientific. IDT, Integrated DNA Technologies, Inc.
